# The relationship between anxious traits and learning about changes in stochasticity and volatility

**DOI:** 10.1371/journal.pcbi.1013646

**Published:** 2025-10-30

**Authors:** Brónagh McCoy, Rebecca P. Lawson

**Affiliations:** 1 Department of Psychology, University of Cambridge, United Kingdom; 2 Department of Forensic and Neurodevelopmental Sciences, Institute of Psychiatry, Psychology and Neuroscience, King’s College London, United Kingdom; Indian Institute of Technology Mandi - Kamand Campus: Indian Institute of Technology Mandi, INDIA

## Abstract

Anxiety is known to alter learning in uncertain environments. Experimental paradigms and computational models addressing these differences have mainly assessed the impact of volatility, with more highly anxious individuals showing a reduced adaptation of learning rate in volatile compared to stable environments. Previous research has not, however, independently assessed the impact of both changes in volatility, i.e., reversals in reward contingency, and changes in stochasticity (noise) in the same individuals. Here, in an original online study (Experiment 1; N = 80) and a pre-registered replication attempt (Experiment 2; N = 160), we use a simple probabilistic reversal learning paradigm to independently manipulate the level of volatility and noise at the experimental level in a fully orthogonal design. We replicate previous studies showing general increases, irrespective of anxiety levels, in positive learning rate (Experiment 1) and negative learning rate (Experiments 1 and 2) for high compared to low volatility, but here only in the context of low noise. Across both experiments, there was an interaction between volatility and noise on behaviour, with more win-stay responses for high compared to low volatility under low noise, but similar or fewer win-stay responses for the same comparison under high noise. The impact of anxious traits presented differently across experiments; in Experiment 1, increases in lose-shift responses in high versus low noise conditions scaled with level of anxious traits, whereas in Experiment 2, there was a full interaction between volatility, noise and anxious traits on win-stay behaviour. These anxiety-related lose-shift or win-stay differences were reflected in their corresponding negative and positive reinforcement learning rate parameters, respectively. Experiment 2 represents a more robust set of results with a larger sample size, balanced gender representation, and extended block order balancing. These findings suggest that changes in both sources of uncertainty - stochasticity and volatility - should be carefully considered when investigating learning and how learning is shaped by anxiety.

## Author summary

Adapting to changes in our environment is a daily endeavour. To do so, humans and animals alike make use of feedback to guide future actions. Uncertainty in the environment can arise from the probabilistic structure inherent in nature (noise) or from an actual change or switch in what is rewarded (volatility). We propose that anxious people respond differently to changes in environmental noise and volatility. Through a combination of computational modelling and behavioural analyses, we demonstrate changes in behaviour that depend on different combinations of noise and volatility, in terms of how much people stick with a previous choice if it was rewarded, or shift to a different option if unrewarded. We also show that anxiety levels play an important role in how people learn under these changeable conditions. Given that anxiety is a common experience for many people in the fast-paced, changing environments of modern society, our modelling approach provides novel insights into the underlying mechanisms at play in the face of different sources of uncertainty, and may help inform interventions aimed at supporting anxious individuals.

## Introduction

Learning in uncertain environments requires appropriate and often significant adaptation. In recent years, researchers have focused on how healthy individuals and those with neuropsychiatric conditions respond to environmental volatility, by probing how individuals adjust their learning based on how changeable the environment is, i.e., how the association between a stimulus and/or action and its subsequent outcome changes over time [1]. People with anxiety disorders often experience symptoms such as greater intolerance of uncertainty [2–5], greater sensitivity to negative events [6], and elevated avoidance tendencies [7,8] compared to healthy controls. These features of anxiety have been linked to difficulties in learning under uncertainty, but the mechanisms by which uncertainty disrupts adaptive learning in anxiety is not fully understood.

To reduce uncertainty, the brain is believed to operate in a Bayesian way, evaluating the statistical structure of the environment, and continuously trying to make sense of the causes of sensory inputs [9–11]. According to reinforcement learning (RL) theory, surprising events lead to prediction errors, which are then used to update beliefs about the structure of the environment [12,13]. Learning rates specify the extent to which prediction errors are incorporated into these updated values. Whether to ascribe surprising outcomes to expected uncertainty – inherent uncertainty about the probabilistic nature of events (i.e., noise) – or to unexpected uncertainty – a change or context switch in the stimulus we’re learning about (i.e., volatility) – is a trade-off we encounter every day [14–18]. Previous literature has made predictions about how learning rates may change under different types of uncertainty [18]. If surprising outcomes are caused by noise, i.e., substantial variability in outcomes, then current actions should be based on the average over the outcomes of many previous actions. This is equivalent to a *lower learning rate for noisier environments*, which puts less weighting on individual outcomes. If surprising outcomes are instead caused by a volatile environment, then greater emphasis should be placed on the most recent events to guide optimal choices, i.e., by *increasing learning rate*. A high learning rate may result in over-learning, with more win-stay (sticking with the same choice after receiving a positive outcome) and lose-shift (switching to a different option after receiving a negative outcome) behaviour, whereas a low learning rate may lead to poor updating and result in slow adaptation to a change in the environment [19]. Many previous studies have reported an increase in learning rate for volatile compared to stable environments [1,20–22]. Sensitivity to positive and negative outcomes, e.g., positive and negative learning rates, has previously been linked to win-stay and lose-shift behaviour, respectively [23], although some research suggests that RL parameters and win-stay/lose-shift behaviour may represent separate, potentially complimentary behavioural strategies [24,25].

Learning under uncertainty also depends on the extent to which learned values of stimuli are used to guide decision-making. This is captured by the value sensitivity (inverse temperature) parameter in RL models, capturing the difference between learned values of options, and is representative of an exploration-exploitation trade-off. Studies have shown that from childhood to adulthood, people become less exploratory in their value-based decision-making, i.e., they are more inclined to exploit learned value differences when making decisions [26]. Better task performance during probabilistic RL tasks has been linked to a more exploitative strategy, indicated by a higher value sensitivity parameter [27,28]. Increased value sensitivity has also been associated with increased pupil size during a RL task [29], suggesting the involvement of the locus coeruleus-noradrenergic (LC-NA) neuromodulatory system [30] in value-based decision-making during learning. A prominent theory in the field, the adaptive gain theory, describes a leading role of the LC-NA system in the explore-exploit trade-off [31]. In a putative phasic mode, phasic LC activity responds to task-related events to optimize performance, occurring alongside a moderate level of tonic activity. In a tonic mode, tonic LC activity takes over as neurons stop responding phasically when utility in the task diminishes, leading to distractibility and poorer performance but also to the exploration of alternative options. This tonic mode may be adaptive by aiding a change in behaviour if either the current task is no longer rewarding or if the environment has changed.

Anxious individuals show reduced adaptability of learning rate to changes in volatility, in threatening [20] and rewarding contexts [19]. In an experiment that posed the potential of a shock, individuals high in trait anxiety, as compared to those with lower anxiety, exhibited a blunted increase in learning rate when moving between a stable and volatile environment (and vice versa), i.e., trait anxiety was associated with a reduced ability to appropriately adapt to a volatile environment [20]. A similar study reported elevated learning rates for negative outcomes in a volatile punishment environment in those with mood and anxiety symptomatology compared to controls [32]. A study on state anxiety, i.e., current anxious arousal as a separate (but associated) construct to trait or chronic anxiety, examined anxiety-associated differences in uncertainty representations, describing how, in a highly volatile and noisy environment, state-anxious individuals exhibit a reduced estimate of volatility, leading to a lower learning rate compared to those with less anxiety [33].

It has recently been proposed that the difference in uncertainty processing in anxious individuals, rather than being associated with volatility, is in fact due to an underlying deficit in estimating stochasticity (noise) [34]. The authors suggest that anxiety primarily disrupts inference about stochasticity but that the learner misinterprets fluctuations due to stochasticity as a signal of change, i.e., volatility. Predictions from Piray & Daw’s model [34] were partly based on data from Huang and colleagues [19], obtained from a change point detection task. In their research, Huang et al. suggested that individuals with high anxiety had difficulty determining if an action was associated with an outcome by chance (noise) or by some statistical regularity in the environment (volatility), indicated by a higher lose-shift rate in people with high compared to low anxiety, i.e., those with high anxiety were more inclined to switch to choosing the other option after receiving negative feedback. In Huang et al., participants performed a visual discrimination task, in which they had to identify a target at one of three locations. Each participant was assigned a target random dot motion coherence direction (30% coherence to either the left or right), and searched through the locations for the assigned target. Each location was associated with either a low, medium, and high reward rate, representing a manipulation of noise levels. At regular intervals a change point occurred, and the reward rate levels were switched across the three locations. As well as the potential for individual differences in perceptive abilities for such visual discrimination tasks, and the need to keep track of three locations, the level of volatility was consistent throughout the task. Within-participant differences due to controlled changes in both noise and volatility levels were therefore not observed in this study. In other mentioned studies, volatility and noise were either fluctuating in a complex, (pseudo-) random manner across the experiment [33], or there was a change from a stable to volatile environment (or vice versa), without any targeted manipulation of noise levels [1,20–22]. Moreover, the research aims of several of these studies were to examine the relationship between behaviour under uncertainty and state anxiety [33] or autism [21,22]. To the authors’ knowledge, few if any studies to date have systematically isolated changes in volatility from changes in noise at the experimental level, and tested how these may be differentially processed by people high in trait anxiety.

In the current study, we use a standard probabilistic reversal learning task to explicitly test how changes in uncertainty impact learning behaviour, by orthogonally manipulating the level of noise and volatility in the environment. Using behavioural measures of accuracy, win-stay and lose-shift responses, alongside hierarchical Bayesian modelling, we examine changes across these different levels of uncertainty and address how specific combinations of noise and volatility levels across experimental blocks affect learning in adults with a range of anxious traits. Our research questions are two-fold: 1) how does increasing noise affect behaviour and learning parameters in the general population and how does it differ from increasing volatility, and 2) are anxious people differentially impacted by changes in noise and volatility? Consistent with prior theoretical work [18,34], we predicted that across all participants, an increase in volatility would engender higher learning rates, and an increase in noise should lead to a lowering of learning rates, with similar shifts in behavioural win-stay and/or lose-shift correlates. However, if highly anxious individuals are more likely to mistake a change in noise for a change in volatility, then we would expect highly anxious people to display higher learning rates in response to an increase in noise, compared to less anxious people. This extends previous empirical work suggesting that anxious people have difficulty determining if an action is associated with an outcome by chance or by some statistical regularity [19], by manipulating volatility and noise in a controlled manner at the individual participant level.

## Experiment 1

### Results

**Fig 1:**
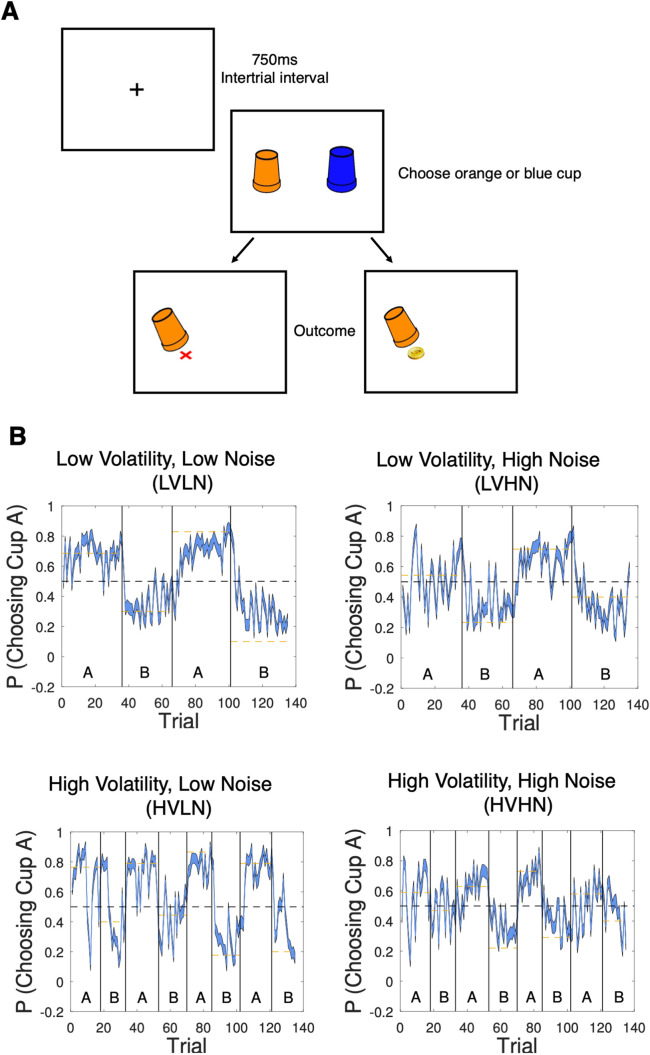
Experimental paradigm and trial-by-trial behavioural responses. A) On each trial, participants were presented with a blue and orange cup and had to choose one of them, receiving either a coin as positive feedback or a red ‘x’ as negative feedback. B) Probability of choosing one of the cups (denoted here as Cup A) across time in the full sample (Experiment 1; N = 80). Vertical lines represent reversals in contingency, with three reversals occurring in the low volatility condition and seven reversals for high volatility. Dashed yellow line represents the underlying true contingency in each miniblock. Trial-by-trial responses are shown for all four experimental conditions. Confidence intervals are ± 1 SEM around the group mean.

Eighty participants completed four blocks (135 trials each) of a probabilistic reversal learning task (Fig 1A), in an orthogonal design with low or high volatility combined with low or high noise. The effects of volatility and noise were assessed by doubling the rate of volatility between low and high volatility conditions (i.e., 3 vs. 7 reversals), and by reducing the level of noise by 10% from low to high noise conditions (see Methods). The reversal structure and averaged trial-by-trial responses for each condition are shown in Fig 1B. On each trial participants had to choose between a blue and an orange cup to try and find the reward (gold coin) hidden underneath. They were instructed to try to choose the cup that gives more reward on average and across time. They were told that the best cup may change over time and they should figure out when to stop choosing one cup and switch to the other. A minimum sample size of 34 participants was estimated by a power analysis based on a reported correlation between level of anxious traits and a volatility-induced shift in learning rate [20] (see Text A in S1 Appendix). This was bolstered to 80 participants to capture any additional effects due to the manipulation of noise levels.

All participants completed the state-trait anxiety inventory (STAI) to capture the extent of anxious traits. Mean STAI trait score was 50.75 ± 10.21 (lowest possible score was 20, highest was 80). As has been demonstrated previously [35], the state and trait components of the STAI were highly correlated (*r* = 0.71, p < .001). Analyses were carried out firstly on the full sample, using the continuous trait domain of the STAI. Participants were then split into low-moderate and high anxiety groups according to their level of anxious traits (N = 28 and N = 27 respectively). Based on a tertile split, STAI trait scores were <=46 for the low-moderate anxiety group and>=54.67 for the high anxiety group. For presentation purposes, we will refer to the low-moderate anxiety group as low ANX, relative to the high ANX group. This distinction between low and high ANX traits was used to ensure greatest separation between groups. This binary category-based group-level approach was established especially for carrying out hierarchical Bayesian modelling (see Methods). Although hierarchical Bayesian modelling has many strengths [36], subject-level learning parameter estimates are prone to “shrink” towards the group-level mean, thereby underestimating group-level variance. We thus split the data into groups based on levels of anxious traits so we could carry out statistics on posterior distributions at the group-level (low/high ANX groups), which does not rely on these shrunken subject-level estimates.

### Data preprocessing

As participants were not instructed to respond quickly, we set a lenient upper cut-off of 4000ms for average reaction times (RTs) to ensure they were not taking breaks within blocks of trials. Average RTs were below the predetermined cut-off of 4000ms for all participants, allowing all data to be included in further analysis. Four participants had an average RT of greater than 2 seconds, the longest of which was 3.13 seconds. Since the environment was highly changeable there was no objective cut-off for accuracy. A lower threshold for exclusion was therefore defined as less than 2.5 SD from the mean overall accuracy (62.24 ± 8.43%), calculated as 41%. No participants surpassed this threshold; four participants scored lower than 50% accuracy, the lowest of which was 46.67%.

### Demographics

The full sample (N = 80) consisted of 61 female participants, 16 males, and 3 responding as neither (non-binary, other, or prefer not to say categories), with a mean age of 24.31 ± 7.67 years. After splitting data into low and high ANX groups, there were no significant group differences in age (low ANX: 24.14 ± 8.39 years, high ANX: 22.44 ± 4.41 years; W = 381.5, p = .959), gender (low ANX: 71.3% female, high ANX: 74.07% female; *X*^2^(4, N = 55) = 4.32, p = 0.365), or education level (binarized for obtaining bachelor degree or higher; low ANX: 53.57%, high ANX: 51.85%; Fisher’s exact test: p = 1).

### Behavioural analysis

#### Baseline effects of noise and volatility on behavioural measures in the full sample.

**Fig 2:**
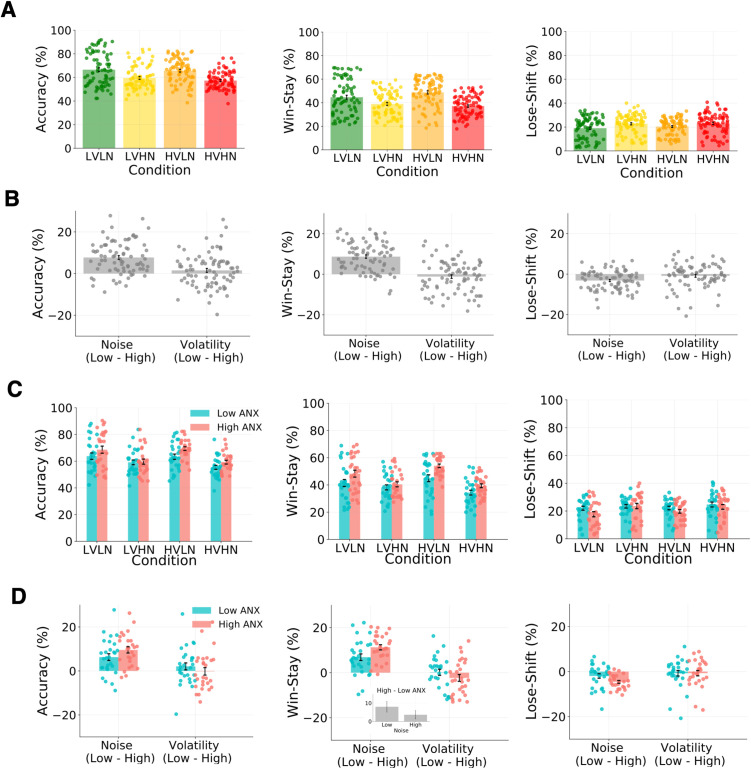
Experiment 1 - Behavioural results. Accuracy, win-stay responses, and lose-shift responses per condition in **(A)** the full sample (N = 80) and **(C)** low (N = 28) and high (N = 27) ANX groups. Differences in these measures for low vs. high noise and low vs. high volatility are shown in **(B)** the full sample and **(D)** low and high ANX groups. **(A, B)** Results demonstrate a visible effect of noise level on behaviour, with better task performance, more win-stay behaviour, and less lose-shift behaviour under low compared to high noise conditions (all p < .001 in full sample). Participants showed more win-stay responses for high compared to low volatility in the context of low noise, and less win-stay behaviour for high compared to low volatility under high noise (p < .001 in full sample). Note that volatility-induced change in the three behavioural measures is minor compared to noise-induced change. **(C, D)** The high ANX group had significantly more win-stay responses than the low ANX group under low (p_bonf _= .015) but not high noise (p_bonf_ = .962) (see inset in **D**). The high ANX group also showed significantly more lose-shift responses in the high compared to low noise conditions (p_bonf _< .001) whereas the low ANX group did not display meaningful noise-related differences in lose-shift behaviour (p_bonf_ = 0.132). LVLN: low volatility with low noise, LVHN: low volatility with high noise, HVLN: high volatility with low noise, HVHN: high volatility with high noise. Error bars are ± 1 SEM.

Average RT and accuracy across the full sample was 808.06 ± 482.55ms and 62.24 ± 8.43%, respectively (see Table 1 and Fig 2A for reported behavioural results; see Fig A in S1 Appendix for RTs). Only significant results are presented in the following analyses. A repeated-measures ANOVA on task accuracy showed a significant main effect of noise level (F(1,79)=79.149, p < .001, η_p_^2^ = 0.500), with greater accuracy in the low compared to high noise conditions. To assess immediate responses to feedback, or reactivity, we looked at the tendency to stick with choosing the same option immediately after receiving positive feedback (win-stay) and to shift responding after negative feedback (lose-shift). For win-stay behaviour, we found a significant main effect of noise (F(1,79)=114.069, p < .001, η_p_^2^ = 0.591), with fewer win-stay responses in high noise blocks. Although not mapping directly onto learning rate, this reduction in win-stay behaviour under high noise suggests that participants interpreted positive outcomes under high noise as being potentially less informative about the actual state of the environment. There was also a volatility*noise interaction (F(1,79)=15.022, p < .001, η_p_^2^ = 0.160), with more win-stay responses for high compared to low volatility in the context of low noise, and less win-stay behaviour for high compared to low volatility in the context of high noise. For lose-shift behaviour we found a significant main effect of noise only (F(1,79)=35.465, p < .001, η_p_^2^ = 0.310), with more shifting under high noise. The combination of fewer win-stay and more lose-shift responses under high noise indicates more shifting behaviour in general under high noise, regardless of feedback.

**Table 1:** Experiment 1 - Behavioural results for the full sample (N = 80). Average accuracy, reaction time (RT), win-stay, and lose-shift behaviour is shown per condition – low volatility with low noise (LVLN), low volatility with high noise (LVHN), high volatility with low noise (HVLN), high volatility with high noise (HVHN). Differences in behavioural measures for low vs. high noise and low vs. high volatility are also displayed. Numbers in brackets represent ±1 SD.

*Measure*	LVLN	LVHN	HVLN	HVHN	Low - High Noise	Low – High Vol
Accuracy (%)	66.52 (13.96)	59.50 (9.88)	65.69 (10.30)	57.26 (7.39)	7.72 (7.71)	1.54 (8.09)
RT (ms)	803 (521)	794 (523)	775 (522)	860 (734)	-38 (342)	-19.38 (448)
Win-stay (%)	44.69 (14.89)	38.94 (10.29)	48.80 (12.05)	37.16 (8.86)	8.69 (7.23)	-1.16 (7.62)
Lose-shift (%)	10.07 (9.14)	22.75 (8.16)	20.44 (6.81)	23.10 (8.71)	-3.18 (4.74)	-0.86 (6.01)

A power analysis on the ability of the experimental design to detect expected changes in win-stay and lose-shift behaviour across volatility conditions is included in the supplement (see Text B in S1 Appendix). Although we had no prior hypotheses about how behaviour under changing noise and volatility levels would be impacted by age or gender, we carried out analyses including age as an additional co-variate and gender as a between-subjects factor, which showed no significant main effects of age or gender nor any interactions between them and volatility and/or noise on task accuracy, win-stay behaviour, or lose-shift behaviour (all p < .1).

#### Effects of noise, volatility, and anxious traits on behavioural measures in the full sample.

**Fig 3:**
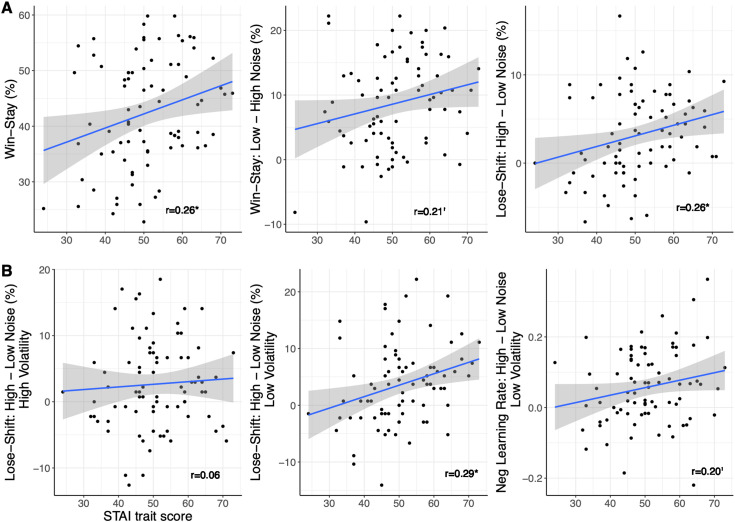
Experiment 1 - Relationship between learning and STAI traits in full sample (N = 80). A) Significant positive correlation between anxious traits and general win-stay behaviour (p = .019; left panel); a positive, trend-only relationship between anxious traits and win-stay behaviour in low compared to high noise conditions (p = .061; middle panel); and a significant positive relationship between anxious traits and lose-shift responses in high compared to low noise conditions (p = .021; right panel). B) No relationship between anxious traits and lose-shift behaviour in high compared to low noise conditions under high volatility (p < .1; left panel), but crucially, a significant positive correlation between anxious traits and lose-shift behaviour in high compared to low noise conditions under low volatility (LVHN – LVLN, p = .009; middle panel); and a positive, trend-only relationship between anxious traits and negative learning rate for the same comparison (LVHN – LVLN, p = .069; right panel). Grey bands represent 95% confidence intervals.

When level of anxious traits was included as a co-variate in the analyses conducted above, a different picture was revealed (see Fig 3 for important relationships). There was no longer a main effect of noise on accuracy, nor any other main or interactive effects of noise or volatility (all p > .1). A positive main effect of anxious traits on accuracy fell just outside the statistical significance threshold (F(1,78)=3.779, p = .056). For win-stay behaviour, there was also no longer a main effect of noise (F(1,78)=0.071, p = .790, η_p_^2^ < 0.001), nor a volatility*noise interaction (F(1,78)=1.170, p = 0.283, η_p_^2^ = 0.015). A noise*anxious traits interaction lay just outside the significance threshold (F(1,78)=3.604, p = .061, η_p_^2^ = 0.044), and there was a significant main effect of anxious traits on win-stay behaviour (F(1,78)=5.691, p = 0.019), with those higher in anxiety displaying more win-stay behaviour in general. For lose-shift behaviour, there was a significant interaction between noise and anxious traits (F = 5.576, p = .021, η_p_^2^ = 0.067), with those higher in anxious traits showing more lose-shift behaviour for high compared to low noise than individuals lower in anxiety. This interaction provides evidence in support of our main research question at the behavioural level, as switch behaviour after negative feedback is thought to reflect the extent of updating on trial-by-trial prediction errors, i.e., negative learning rate. Taken together, these findings suggest that anxious traits play a pivotal role in behavioural responses in this dynamic environment, when learning from positive outcomes in general and when learning from negative outcomes under changing noise levels.

#### Effects of noise, volatility, and low/high ANX group on behavioural measures.

Average RT and accuracy were 914.95 ± 628.75ms and 60.42 ± 7.80% for the low ANX group, and 721.78 ± 244.20ms and 64.25 ± 7.50% for the high ANX group. There was no difference in which condition participants received first (LVLN, LVHN, HVLN, or HVHN) across ANX groups (*X*^2^ (3,55) = 3.4, p = 0.337). An analysis of task accuracy showed a significant main effect of noise level (F(1,53)=58.74, p < .001, η_p_^2^ = 0.51; see Table 2 and Fig 2B), with higher accuracy in the low compared to high noise conditions, as was observed in the full sample analysis. There were no other main effects or interactions (all p > .1). However, the best fitting model according to a Bayesian repeated-measures ANOVA included main effects of both noise and ANX group (BF = 7.14, R^2^ = 0.521, 95% CI=[0.436, 0.595]), with the high ANX group exhibiting higher accuracy in general regardless of volatility or noise level, closely followed by a noise only model (BF = 6.20, R^2^ = 0.514, 95% CI=[0.425, 0.587]). This suggests that both noise and ANX level played an independent role in task performance.

**Table 2:** Experiment 1 - Behavioural results for low ANX (N = 28) and high ANX (N = 27) groups. Average accuracy, reaction time (RT), win-stay, and lose-shift behaviour is shown per condition. Differences in behavioural measures for low vs. high noise and low vs. high volatility are also displayed. Numbers in brackets represent ±1 SD.

	*Low ANX*					
***Measure***	LVLN	LVHN	HVLN	HVHN	Low - High Noise	Low - High Vol
Accuracy (%)	63.78 (12.64)	59.13 (9.36)	63.36 (10.32)	55.40 (7.85)	6.31 (8.05)	2.08 (8.42)
RT (ms)	888 (516)	939 (667)	893 (735)	939 (1017)	-49 (444)	-2 (643)
Win-stay (%)	41.43 (13.52)	38.20 (9.80)	44.92 (13.22)	34.37 (9.33)	6.89 (7.76)	0.17 (7.14)
Lose-shift (%)	22.01 (7.48)	23.39 (6.57)	22.17 (6.71)	24.68 (9.28)	-1.94 (5.27)	-0.73 (6.56)
	***High ANX***					
***Measure***	LVLN	LVHN	HVLN	HVHN	Low - High Noise	Low - High Vol
Accuracy (%)	68.64 (13.84)	59.67 (9.88)	69.44 (6.98)	59.26 (7.19)	9.57 (7.29)	-0.19 (8.78)
RT (ms)	658 (245)	675 (321)	689 (322)	865 (483)	-97 (245)	-111 (314)
Win-stay (%)	48.29 (13.15)	40.44 (9.49)	54.05 (6.80)	39.37 (7.35)	11.26 (5.89)	-2.35 (7.83)
Lose-shift (%)	17.67 (9.59)	23.59 (10.01)	19.73 (6.61)	22.96 (8.82)	-4.58 (3.14)	-0.71 (6.15)

Assessing win-stay behaviour, we found a significant main effect of noise (F(1,53)=94.99, p < .001, η_p_^2^ = 0.642), with more win-stay responses for low compared to high noise conditions. As in the full sample, there was a volatility*noise interaction (F(1,53)=17.43, p < .001, η_p_^2^ = 0.247), with more win-stay responses in high compared to low volatility in low noise conditions, and less win-stay behaviour in high compared to low volatility in high noise conditions. There was also a significant noise*ANX group interaction (F(1,53)=5.51, p = .023, η_p_^2^ = 0.094), with a greater decrease in win-stay behaviour in low vs. high noise conditions in the high compared to low ANX group, i.e., a steeper reduction in the high ANX group in response to higher noise. Post-hoc comparisons showed significantly more win-stay responses for the high compared to low ANX group under low noise (M ± SE = 7.99 ± 2.55, t = 3.13, p_bonf_ = .015) but not under high noise (M ± SE = 3.62 ± 2.55, t = 1.42, p_bonf_ = .962). Finally, there was also a main effect of ANX group, with more win-stay behaviour in general in the high compared to low ANX group (F(1,53)=5.97, p = .018, η_p_^2^ = 0.101). A Bayesian repeated-measures ANOVA confirms these findings on win-stay behaviour but also includes a main effect of volatility in the best fitting model, i.e., main effects of noise, ANX group, and volatility, as well as volatility*noise and noise*ANX group interactions (BF = 13.491, R^2^ = 0.638, 95% CI=[0.574, 0.688]).

For lose-shift behaviour there was a significant main effect of noise, with more lose-shift responses in high compared to low noise conditions (F(1,53)=30.83, p < .001, η_p_^2^ = 0.368). There was also a significant noise*ANX group interaction (F(1,53)=5.03, p = .029, η_p_^2^ = 0.087), with less lose-shift behaviour for the high compared to low ANX group under low noise conditions, but more similar behaviour between ANX groups under high noise conditions. Post-hoc comparisons revealed significantly more lose-shift responses in the high compared to low noise conditions in the high ANX group (M ± SE = 4.58 ± 0.84, t = 5.46, p_bonf _< .001) with no meaningful difference in this measure for the low ANX group (M ± SE = 1.94 ± 0.82, t = 2.36, p_bonf_ = 0.132). These anxiety-related differences in lose-shift behaviour mirror those seen for win-stay behaviour; compared to less anxious people and in the context of low noise, those with higher anxiety stick with the same choice more after receiving positive feedback and shift to the other option less after receiving negative feedback, i.e., more stay behaviour in general. In line with the findings of Huang and colleagues [19], lose-shift responding was driven by greater sensitivity to the level of noise in more anxious participants. Although a Bayesian version of the analysis includes noise only as the best fitting model (BF = 9.95, R^2^ = 0.576, 95% CI=[0.500, 0.635]), there was also evidence for a model including main effects of noise and ANX group, and a noise*ANX group interaction (BF = 4.36, R^2^ = 0.581, 95% CI=[0.497, 0.643]), and for a model with just main effects of noise and ANX group (BF = 4.36, R^2^ = 0.575, 95% CI=[0.489, 0.639]). An overview of low and high ANX groups analyses based on a median split is also available in the supplement (see Fig B in S1 Appendix).

### Hierarchical Bayesian model analysis and model validation

Six learning models were fit to each condition for (1) the full sample and (2) separate high and low ANX groups, and the model with the lowest LOOIC value was selected for further analysis for each of these groups (see Table A and Table B in S1 Appendix). The winning model was the fictitious update reward-punish model with indecision point (FU-RP-IP). For the full sample, the FU-RP-IP model fit the data best in three out of four models, and for the high and low ANX groups, it was the best fit in seven out of eight models. There was one instance, the LVHN condition, where a very similar model without the indecision point was the best fit to the data. Here we present results from the FU-RP-IP model. An analysis of the model without indecision point also showed a similar pattern of results and group differences as those presented here and does not alter our conclusions (see Fig C and Table C in S1 Appendix). Model validation of the winning ANX group models were carried out using posterior predictive checks, to ensure that the model mimics the data sufficiently well, i.e., validate that the model can recapture actual behaviour (see Fig D in S1 Appendix). Furthermore, using two different simulation methods we simultaneously demonstrate both adequate parameter recovery of the ANX group models and the constraining nature of hierarchical group-level parameter distributions on individual-level parameters (see Text C and Fig E in S1 Appendix).

### Volatility and noise condition differences in group-level learning parameters of the full sample

**Fig 4:**
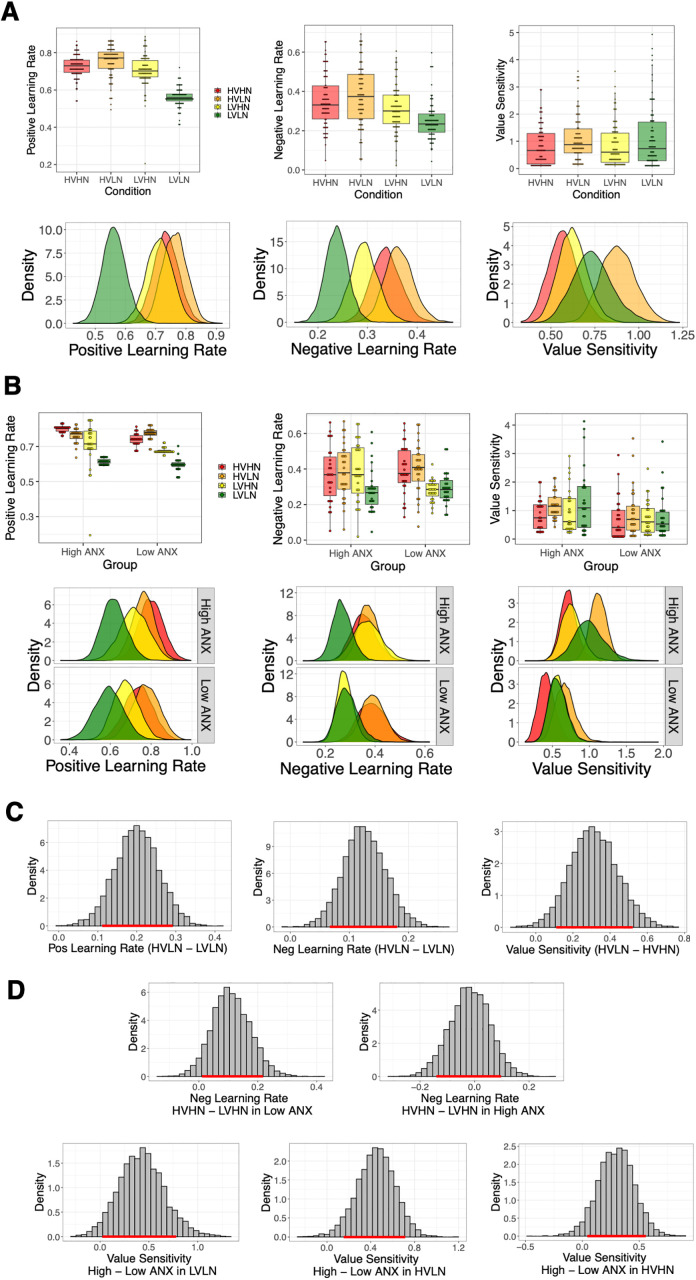
Experiment 1 - Individual-level and group-level model parameters of the winning hierarchical Bayesian model. **(A**) Model parameters of the full sample (N = 80), showing median individual-level parameters (upper panel) and group-level posterior distributions (lower panel). The least uncertain LVLN condition (in green) can be seen to engender a reduced positive learning rate compared to both HVLN (in orange) and LVHN (in yellow), i.e., there is both a volatility- and noise-induced difference in positive learning rate. A similar volatility-induced increase in negative learning rate can also be seen from LVLN to HVLN. **(B)** Model parameters for the low (N = 28) and high (N = 27) ANX groups. Crucially, negative learning rate for the LVHN condition (in yellow) overlaps with the LVLN condition in the low ANX group, i.e., distributions are similar for high versus low noise under low volatility, but is shifted to higher values and overlaps the HVHN condition in the high ANX group. Value sensitivity distributions are higher in general for high compared to low ANX groups. Note that analyses of condition differences according to ANX group are carried out on group-level distributions only. **(C)** Full sample group-level condition differences in three model parameters; with a higher positive learning rate and higher negative learning rate in high compared to low volatility under low noise (left and middle panel, respectively) and greater value sensitivity for low compared to high noise under high volatility (right panel). **(D)** Group-level distribution differences for low and high ANX groups. The LVHN condition is significantly lower than HVHN in the low ANX group (left panel) whereas there is full overlap of these conditions in the high ANX group (right panel). ANX group differences are seen in the value sensitivity parameter, with higher values for the high ANX group in each of the LVLN, HVLN, and HVHN conditions (lower panel). Red bar across the x-axis represents the 89% highest density interval (HDI); HDIs that don’t overlap zero signify a meaningful difference.

Median and standard deviation values of posterior distributions are presented in Table D in S1 Appendix, with posterior group-level distribution plots shown in Fig 4A (also see Fig F in S1 Appendix). For all comparisons between group- and condition-level posterior distributions, we use the highest density interval (HDI), with any interval not crossing zero indicating a meaningful difference between distributions (see Methods). We also report p_dir_, the probability of direction of the difference (positive or negative), and the region of practical equivalence (ROPE), with p_dir _> 95%, and < 2.5% of the posterior falling within the ROPE, considered to be good indictors of a meaningful effect. The positive learning rate parameter was lowest for the LVLN condition, with significantly higher values in the HVLN (HDI = [0.11, 0.29], p_dir_ = 100%, 0.02% inside the ROPE) and LVHN (HDI = [0.06, 0.24], p_dir_ = 99.60%, 0.27% inside the ROPE) conditions, i.e., there was an increase in positive learning rate due to both volatility and noise (see Table 3). This volatility-induced increase was expected based on previous studies [1,20], however, the noise-driven increase was opposite to the hypothesised direction. There was also a volatility-related increase in negative learning rate from LVLN to HVLN (HDI = [0.07, 0.18], p_dir_ = 99.97%, 0.00% inside the ROPE), and a noise-related increase in the value sensitivity parameter from the HVHN to HVLN condition (HDI = [0.11, 0.52], p_dir_ = 99.37%, 0.42% inside the ROPE). Finally, there was a significant increase in both positive and negative learning rate when comparing the most uncertain (HVHN) to least uncertain (LVLN) conditions (positive: HDI = [0.09, 0.27], p_dir_ = 99.80%, 0.13% inside the ROPE; negative: HDI = [0.04, 0.16], p_dir_ = 99.68%, 0.15% inside the ROPE). There were no other significant differences in pairwise-comparisons of parameter distributions (all HDIs overlapped 0). Overall, we found expected increases in both positive and negative learning rate during more volatile conditions, but this occurred under low noise only. There was an unexpected increase in positive learning rate under higher noise, with a noise-driven decrease detected in the value sensitivity parameter instead, i.e., higher noise made people less sensitive to value differences between the options and more exploratory in their choices.

**Table 3:** Experiment 1 – Comparisons between Bayesian model group-level posterior distributions across conditions. The highest density interval (HDI) represents the 89% probability region of the difference probability distribution. Intervals that do not overlap zeros indicate a meaningful difference between distributions, i.e., between conditions.

Model parameter	Between-condition HDI				
	LVLN - LVHN	HVLN - LVLN	HVHN - LVHN	HVLN - HVHN	LVLN - HVHN
Pos. learning rate	-0.24 -0.06*	0.11 0.29*	-0.08 0.11	-0.07 0.12	-0.27 -0.09*
Neg. learning rate	-0.11 0.001^§^	0.07 0.18*	-0.02 0.11	-0.04 0.09	-0.16 -0.04*
Value sensitivity	-0.11 0.32	-0.09 0.38	-0.24 0.13	0.11 0.52*	-0.06 0.38
Indecision point	-0.10 0.09	-0.07 0.14	-0.02 0.16	-0.14 0.06	-0.18 0.03

### Differences in group-level learning parameters for low and high ANX groups

Group-level posterior distribution plots are shown in Fig 4B (also see Fig G in S1 Appendix). Median and standard deviation values of posterior distributions of each ANX group, condition and parameter are shown in Table E in S1 Appendix. Comparing group-level parameters for each condition, there was a significant ANX group difference in the value sensitivity parameter for the LVLN (HDI=[0.03,0.78], p_dir_ = 97.25%, 1.35% inside the ROPE), HVLN (HDI=[0.16,0.71], p_dir_ = 99.12%, 0.27% inside the ROPE) and HVHN conditions (HDI=[0.05,0.55], p_dir_ = 96.57%, 1.40% inside the ROPE), with greater sensitivity for high compared to low ANX. There was no such difference in the LVHN condition (HDI=[-0.10, 0.47], p_dir_ = 85.78%, 4.68% inside the ROPE). See Table 4 for all group and condition comparisons. These findings suggest a more exploitative choice strategy in general in the high ANX group, with choice strategy of the low and high ANX groups being most similar in the LVHN condition.

**Table 4:** Experiment 1 - Differences in ANX groups model parameter posterior distributions. 89% highest density intervals (HDI) on group and condition parameter posterior distribution differences, showing the lower and upper bounds of the 89% HDI. HDI that does not overlap 0 indicates a meaningful difference between distributions. Between-group HDI per condition (top), and between-condition HDI for each group (middle: Low ANX, bottom: High ANX).

	Between-group HDI: High – Low ANX				
**Model parameter**	LVLN	LVHN		HVLN	HVHN
Pos. learning rate	-0.12 0.17	-0.10 0.20		-0.15 0.12	-0.08 0.21
Neg. learning rate	-0.11 0.07	-0.01 0.19		-0.12 0.08	-0.15 0.08
Value sensitivity	**0.03 0.78** ^*^	-0.18 0.47		0.16 0.71*	**0.05 0.55** ^*^
Indecision point	-0.13 0.25	-0.14 0.11		-0.10 0.25	0.01 0.36*
	**Between-condition HDI: Low ANX**				
**Model parameter**	LVLN - LVHN	HVLN - LVLN	HVHN - LVHN	HVLN - HVHN	LVLN - HVHN
Pos. learning rate	-0.27 0.09	0.02 0.33*	-0.09 0.20	-0.11 0.18	-0.30 0.01
Neg. learning rate	-0.09 0.12	-0.004 0.20^§^	0.01 0.22*	-0.11 0.12	-0.21 0.01
Value sensitivity	-0.33 0.33	-0.18 0.39	-0.41 0.10	-0.01 0.54	-0.11 0.42
Indecision point	-0.25 0.16	-0.13 0.27	-0.17 0.13	-0.17 0.20	-0.22 0.14
	**Between-condition HDI: High ANX**				
**Model parameter**	LVLN - LVHN	HVLN - LVLN	HVHN - LVHN	HVLN - HVHN	LVLN - HVHN
Pos. learning rate	-0.27 0.08	0.02 0.27*	-0.07 0.22	-0.11 0.18	-0.32 -0.05*
Neg. learning rate	-0.24 0.02	0.02 0.20*	-0.14 0.10	-0.08 0.12	-0.19 0.01
Value sensitivity	-0.25 0.71	-0.26 0.47	-0.35 0.22	0.16 0.66*	-0.11 0.62
Indecision point	-0.15 0.23	-0.10 0.22	0.04 0.35*	-0.24 0.08	-0.33 0.05

Comparing conditions in each ANX group separately, there was a significantly higher positive learning rate in the HVLN compared to LVLN condition in both low (HDI=[0.02,0.33], p_dir_ = 96.53%, 1.33% inside the ROPE) and high ANX groups (HDI= [0.02,0.27], p_dir_ = 96.45%, 1.43% inside the ROPE), reflecting findings from the full sample model in both groups. For the same HVLN versus LVLN comparison, there was also a significant shift in negative learning rate in the high ANX group (HDI=[0.02,0.20], p_dir_ = 96.90%, 1.15% inside the ROPE). Although not significant, the low ANX group showed a very similar shift (see Table 4). Notably, the low ANX group showed a significantly higher negative learning rate in the HVHN compared to LVHN condition (HDI=[0.01,0.22], p_dir_ = 95.95%, 1.93% inside the ROPE), but this difference was not exhibited in the high ANX group, with overlapping HVHN and LVHN distributions (HDI=[-0.14,0.10], p_dir_ = 81.68%, 5.67% inside the ROPE; see Fig 4B). This suggests that highly anxious people employ a similar negative learning rate under both high noise conditions, without making an adjustment for the change in volatility. Finally, the high ANX group showed significantly higher value sensitivity in the HVLN compared to HVHN condition (HDI=[0.16,0.66] p_dir_ = 99.32%, 0.45% inside the ROPE), i.e., under high volatility, those with high ANX showed substantially more exploitation of learned value differences for low compared to high noise.

### Relationship between anxious traits and noise-related changes in negative learning rate in the full sample

Based on our hypothesis that more highly anxious people would display higher learning rates in response to an increase in noise compared to less anxious people (for a fixed level of volatility), as well as the reported differences in lose-shift behaviour and the negative learning rate parameter across conditions in both the full sample and ANX groups analyses, we assessed whether there was a relationship between anxious traits in the full sample and noise-related changes in negative learning rate (see Fig 3B). A post-hoc analysis on the reported relationship between anxious traits and noise-related changes in lose-shift behaviour applied across the different volatility levels revealed that this relationship was strongly driven by the low volatility condition (LVHN – LVLN; r = .292, p = .009), i.e., noise-related lose-shift behaviour scaled with levels of anxious traits under low but not high volatility. Despite known issues of “shrinkage” in hierarchical models where individual-level estimates are pulled towards the group-level mean, thus reducing the variance between participants, we followed up on this comparison in the negative learning rate parameter. Although not passing the significance threshold, there was also an indication of higher negative learning rates in the LVHN compared to LVLN condition with increasing anxious traits (r = 0.20, p = .069). Taken together, these associations based on the full sample largely confirm those found in the ANX group-level analyses and extend those findings to demonstrate the effect of anxious traits and noise on learning model parameters.

## Interim discussion

Findings from Experiment 1 revealed general increases, irrespective of anxiety levels, in positive learning rate for high compared to low volatility conditions, but only in the context of low noise. An increase in positive learning rate for high versus low noise was the opposite to what was hypothesized, with previous literature predicting a decrease in learning rate when noise is increased [18,34]. This positive learning rate finding, however, was not reflected in our analyses of win-stay behaviour; this showed a significant drop in win-stay responses under high noise. This mismatch between win-stay and positive learning rate adaptations to high noise, along with a reduction in the value sensitivity parameter under high noise, could point towards the absorption of the win-stay effect by different parameters that are included in our model but may not be present in other models in previous research. When low volatility was combined with high noise, anxious individuals displayed negative learning rates similar to high volatility with high noise, whereas those lower in anxiety showed the previously reported negative learning rate increase from low to high volatility. Within-individual increases in lose-shift responses for high versus low noise conditions scaled with level of anxious traits, and this was prominent mainly in the context of low volatility. We furthermore found that people with higher anxious traits were more accurate overall and utilized a more exploitative decision-making strategy in this dynamic environment. Together, these findings suggest a complex interplay between volatility and noise on behaviour, and an important role of anxiety in how people respond to these forms of environmental uncertainty. Nevertheless, given the relatively small sizes of the low and high ANX group for an online study on trait anxiety, as well as the lack of balanced gender representation, with the study consisting of 76% females, we conducted an additional online replication study in a larger sample of participants with a more balanced gender ratio.

## Experiment 2

We carried out a preregistered replication study (N = 160, 20–50 years; see Text D in S1 Appendix for power analysis). The experiment was performed similarly to Experiment 1, with two additions: i) four new block orders were introduced (making eight in total), to ensure sequences of block order were fully balanced across volatility and noise conditions (see Methods), and ii) sporadic attention checks were introduced twice per block, with participants responding to a simple question about which coloured cup they just chose or the outcome they just received. As per the preregistration, these attention checks were not used as grounds for exclusion but were further assessed in an exploratory analysis on their relationship to learning rate. As in Experiment 1, anxious traits were included as a continuous measure in analyses of the full sample, and participants were again split into low and high ANX groups for direct comparison with results from the Bayesian models, but this time on the basis of a median split to ensure full use of the data.

### Data preprocessing

For Experiment 2, more stringent preregistered thresholds were applied to ensure participants were responding appropriately within blocks. We excluded participants who: i) failed to respond within 10 seconds (too slowly) on more than 5% of all trials, i.e., on more than 27 trials, ii) responded within 150 ms (too quickly) on more than 5% of all trials, iii) chose one of the coloured cups (blue or orange) on less than 10% of trials, i.e., less than 54 trials, and iv) chose one side of the screen (left or right) on less than 10% of trials. Overall, no participants met the first criterion, and seven participants were excluded under the second criterion, for responding too quickly on too many trials. No participants were excluded on the basis of their colour or screen side choices. A technical issue led to data exclusion of one additional participant.

### Demographics

In the full analysed sample (N = 152), there were 77 female participants, 72 males, and 3 responding as neither (non-binary, other, or prefer not to say categories), with a mean age of 33.43 ± 9.02 years.

These demographics display a more balanced female to male representation and older participants compared to participants in Experiment 1. After splitting data into low (N = 78) and high ANX (N = 74) groups, based on a median STAI-T score of 45, there were no group differences in age (low ANX: 33.53 ± 9.21 years, high ANX: 33.32 ± 8.88 years; W = 2923, p = .892), gender (low ANX: 48.70% female/ 48.70% male, high ANX: 52.70% female/ 45.90% male; *X*^2^(3, N = 152) = 1.13, p = .770), or education level (binarized for obtaining bachelor degree or higher; low ANX: 74.36%, high ANX: 66.22%; Fisher’s exact test: p = .29).

### Effects of noise, volatility, and anxious traits on behavioural measures in the full sample

**Fig 5:**
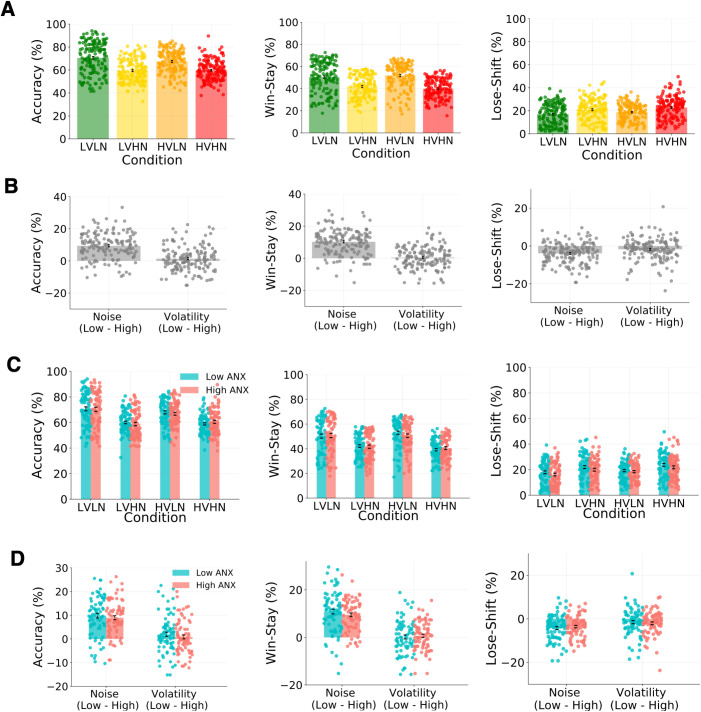
Experiment 2 – Behavioural results. Accuracy, win-stay, and lose-shift responses per condition in **(A)** the full sample (N = 152) and **(C)** low (N = 78) and high (N = 74) ANX groups. Differences in these measures for low vs. high noise and low vs. high volatility are shown in **(B)** the full sample and **(D)** low and high ANX groups. **(A, B)** Main effects of noise on behaviour seen in Experiment 1 (see Fig 2) were replicated, with greater task performance, more win-stay behaviour, and less lose-shift behaviour under low compared to high noise conditions (all p < .01 in full sample).There was a volatility*noise interaction on accuracy (p = .001), with higher accuracy in low compared to high volatility under low noise, but similar levels under high noise across the volatility conditions. There was also a volatility*noise interaction on win-stay behaviour (p = .001), with more win-stay for high compared to low volatility under low noise but less win-stay for high compared to low volatility under high noise, replicating Experiment 1. There were more lose-shift responses under high compared to low volatility (p < .001). **(C, D)** A three-way interaction between volatility, noise and ANX group on win-stay behaviour was found (p = .022). Although both ANX groups show more win-stay responses for low compared to high noise conditions (also captured across the full sample), this effect is heightened in the low ANX group, whereas the high ANX group show elevated win-stay behaviour for low vs. high volatility, compared to the low ANX group. Note that volatility-induced change in win-stay behaviour is minor (also seen in the full sample) compared to noise-induced change, also seen in Experiment 1. Error bars represent ± 1 SEM.

Analysis of task behaviour before accounting for anxious traits is presented in the Supplement (see Text E and Fig H in S1 Appendix). Repeated-measures ANOVAs were performed on accuracy, win-stay, and lose-shift responses across all participants (see Table 5 and Fig 5A), with level of anxious traits included as a co-variate. Logistic mixed-effects (LME) regression models on trial-by-trial behaviour were also carried out to increase the power and flexibility of the analyses. There was a main effect of noise on accuracy (F(1,150) = 19.23, p < .001, η_p_^2 ^= .114), a main effect of volatility (F(1,150) = 6.75, p = .010, η_p_^2 ^= .043), and an interaction between volatility and anxious traits (F(1,150) = 4.24, p = .041, η_p_^2 ^= .027). These effects demonstrate reduced accuracy for high compared to low noise and for high compared to low volatility, along with increased performance for those with higher anxious traits under high volatility, but reduced performance for higher anxious traits under low volatility. A LME regression model on trial-by-trial correct/incorrect responses, which included a random intercept for subject and random slopes for noise, volatility and their interaction, confirmed the negative main effect of noise (b = -.252, z = 7.04, p < .001). However, the model also showed a positive main effect of volatility (b = .110, z = 2.41, p = .016), i.e., effect in opposite direction to the main effect of the ANOVA, and a negative volatility*noise interaction (b = -.101, z = 2.08, p = .037), highlighting similar accuracy under high noise regardless of volatility level but higher accuracy for high compared to low volatility under low noise. The change in direction of the main effect of volatility is likely due to this interaction. Taken together, these results on task performance capture effects of volatility, noise, and their interaction that were not present in Experiment 1.

**Table 5:** Experiment 2 - Behavioural results for the full sample (N = 152). Average accuracy, reaction time (RT), win-stay, and lose-shift behaviour is shown per condition – low volatility with low noise (LVLN), low volatility with high noise (LVHN), high volatility with low noise (HVLN), high volatility with high noise (HVHN). Differences in behavioural measures for low vs. high noise and low vs. high volatility are also displayed. Numbers in brackets represent ±1 SD.

*Measure*	LVLN	LVHN	HVLN	HVHN	Low - High Noise	Low - High Vol
Accuracy (%)	70.54 (13.78)	59.44 (9.82)	67.45 (9.91)	59.73 (8.84)	9.41 (7.97)	1.40 (7.46)
RT (ms)	656 (582)	667 (390)	608 (277)	621 (260)	-12 (325)	48 (324)
Win-stay (%)	50.45 (14.22)	41.94 (10.14)	51.76 (10.14)	39.82 (8.95)	10.22 (7.75)	0.40 (6.57)
Lose-shift (%)	17.04 (9.26)	20.96 (9.62)	18.81 (7.35)	22.75 (9.46)	-3.93 (5.39)	-1.78 (5.79)

**Fig 6:**
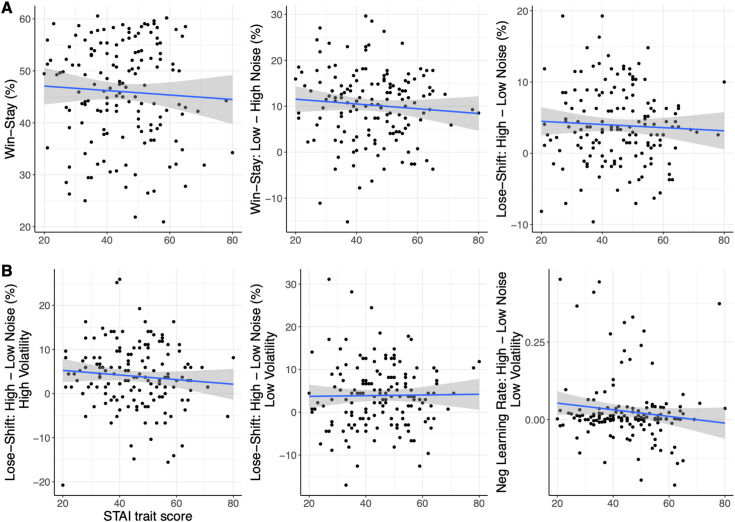
Experiment 2 (N = 152) – Replication attempt. Relationships between learning measures and anxious traits from Experiment 1 (Fig 3) were not replicated. All associations were insignificant (p > .1).

**Fig 7:**
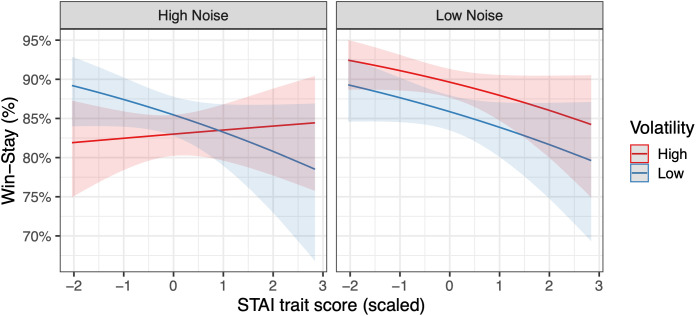
Experiment 2 – Interaction between volatility, noise and anxious traits on win-stay behaviour. There were similar reductions in win-stay behaviour for higher anxious traits under LVLN, LVHN, and HVLN conditions, but no change in win-stay behaviour according to anxious traits in the HVHN condition. This three-way interaction was verified by a repeated-measures ANOVA (p = .039) and LME regression model on stay/switch behaviour (p = .014).

For win-stay and lose-shift behaviour, several of the effects of anxiety in Experiment 1 were not replicated (see Fig 3 for Experiment 1; Fig 6 for Experiment 2). Here, similar to Experiment 1, participants displayed more win-stay behaviour under low compared to high noise (F(1,150)=27.41, p < .001, η_p_^2 ^= .155). There was also a similar interaction between volatility and noise, with more win-stay responses for high compared to low volatility under low noise, and less win-stay behaviour for high compared to low volatility under high noise (F(1,150)=8.38, p = .004, η_p_^2 ^= .053). In distinction to Experiment 1, there was an interaction between volatility, noise and anxious traits (F(1,150)=4.33, p = .039, η_p_^2 ^= .028), with similar reductions in win-stay behaviour for higher anxious traits under LVLN, LVHN, and HVLN combinations, but virtually no change in win-stay behaviour with increasing anxious traits in the HVHN condition (see Fig 7). A LME regression model with stay/switch as the dependent variable, which included subject as a random intercept, and noise, volatility, and their interaction as random slopes, confirmed this interaction between volatility, noise and anxious traits on win-stay behaviour (b = 0.219, z = 2.45, p = 0.014). For lose-shift behaviour, there was more shifting under high noise only (F(1,150)=8.66, p = .004, η_p_^2 ^= .055), with no other effects or interactions with anxious traits. This lack of interaction was corroborated by a LME regression model. These findings stand in contrast to the anxiety-related findings in lose-shift behaviour seen in Experiment 1.

As in Experiment 1, exploratory analyses were also carried out to check for effects of age and gender as co-variates. LME regression models, which allowed for interactions with anxious traits, revealed a significant role of gender and age on behaviour in this task; notably, a three-way interaction between volatility, anxious traits, and gender on win-stay behaviour (b = -0.303, z = 2.17, p = .030; see Fig I in S1 Appendix), and a three-way interaction between noise, anxious traits, and age on lose-shift behaviour (b = 0.076, z = 2.09, p = .036; see Fig J in S1 Appendix). See Text F in S1 Appendix for further details on these interactions and other findings from the gender and age regression analyses.

Overall, although anxious traits do contribute to behavioural differences according to the nature of uncertainty across the task, behavioural findings from Experiment 2 differ from those found in Experiment 1 - namely, in Experiment 2 there was an interaction between volatility, noise and anxious traits on win-stay behaviour, and here we demonstrate that gender and age also interact with anxiety when responding to changes in environmental uncertainty.

### Differences in group-level reinforcement learning parameters of the full sample

**Fig 8:**
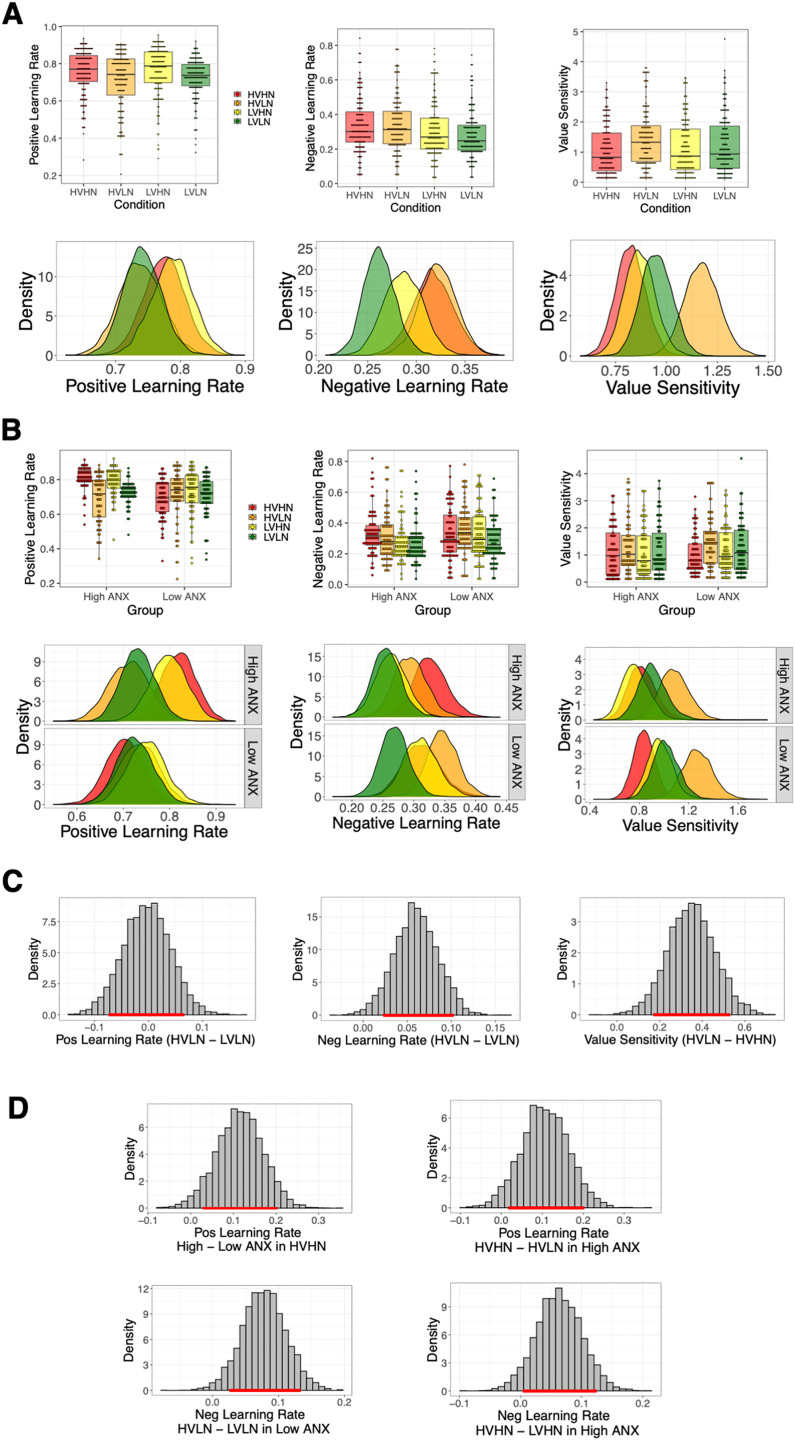
Experiment 2 – Individual-level and group-level model parameters of the winning hierarchical Bayesian model. **(A)** Model parameters of the full sample (N = 152), showing median individual-level parameters (upper panel) and group-level posterior distributions (lower panel). As in Experiment 1 (see Fig 4), there was a volatility-induced higher negative learning rate in the HVLN compared to LVLN condition. A higher value sensitivity was also seen in the HVLN condition compared to both the LVLN and HVHN conditions. **(B)** Model parameters for the low (N = 78) and high (N = 74) ANX groups. Note that the overlap of negative learning rate LVLN (in green) and LVHN (in yellow) distributions according to ANX group is not the same as was found in Experiment 1. **(C)** The same full sample group-level condition difference distributions as seen in Fig 4C are shown. Unlike in Experiment 1, there was no meaningful difference between LVLN and HVLN in positive learning rate in Experiment 2 (left panel). Like in Experiment 1, however, negative learning rate was significantly higher in the HVLN compared to LVLN condition, and value sensitivity was also higher in the HVLN compared to HVHN condition. **(D)** Group-level distribution differences for low and high ANX groups. Anxiety-based differences were generally found in be different in Experiment 2; here we present meaningful ANX-group level differences in Experiment 2. For positive learning rate, the high ANX group had a higher positive learning rate in the HVHN condition than the low ANX group (upper left panel). In the high ANX group only, positive learning rate was also found to be higher in the HVHN compared to HVLN condition (upper right panel). In the low ANX group, negative learning rate was found to be higher in the HVLN than LVLN condition (lower left panel), replicating Experiment 1 (see Fig 4D). Finally, in contrast to Experiment 1, negative learning rate was higher in the HVHN than LVHN condition in the high ANX group (lower right panel). Red bar across the x-axis represents the 89% highest density interval (HDI); HDIs that don’t overlap zero signify a meaningful difference.

The winning Bayesian hierarchical model from Experiment 1 were applied to data from Experiment 2; the fictitious update reward-punish model with indecision point (FU-RP-IP). Group-level posterior distribution plots are shown in Fig 8A (also see Fig K in S1 Appendix for posteriors of the indecision point parameter). Median and standard deviation values of posterior distributions of each condition and parameter are displayed in Table F in S1 Appendix. There were no significant differences in positive learning rate between conditions (see Table 6). There was a higher negative learning rate in the HVLN than LVLN condition (HDI = [0.02, 0.10], *p*_dir _= 99.10%, 0.42% inside the ROPE). There was also higher value sensitivity in the HVLN condition compared to both the LVLN (HDI = [0.04, 0.40], *p*_dir _= 97.28%, 1.13% inside the ROPE) and HVHN conditions (HDI = [0.17, 0.53], *p*_dir _= 99.88%, 0.12% inside the ROPE).

**Table 6:** Experiment 2 - Comparisons between Bayesian model group-level posterior distributions across conditions. The highest density interval (HDI) represents the 89% probability region of the difference probability distribution. Intervals that do not overlap zeros indicate a meaningful difference between distributions, i.e., between conditions.

Model parameter	Between-condition HDI				
	LVLN - LVHN	HVLN - LVLN	HVHN - LVHN	HVLN - HVHN	LVLN - HVHN
Pos. learning rate	-0.11 0.02	-0.07 0.07	-0.08 0.06	-0.11 0.04	-0.10 0.03
Neg. learning rate	-0.06 0.02	0.02 0.10*	-0.02 0.08	-0.04 0.05	-0.10 -0.01*
Value sensitivity	-0.09 0.25	0.04 0.40*	-0.22 0.12	0.17 0.53*	-0.04 0.30
Indecision point	-0.06 0.05	0.02 0.13*	0.05 0.15*	-0.08 0.02	-0.15 -0.05*

### Behaviour and learning parameters in low and high anxiety groups

Behavioural analyses based on a binary median split on ANX traits - into low ANX (N = 78) and high ANX (N = 74) groups - showed largely similar results to the full sample analyses with a continuous STAI trait measure (see Fig 5C and Table G in S1 Appendix for behavioural results). The three-way interaction between volatility, noise and ANX group on win-stay behaviour was significant according to a repeated-measures ANOVA (F(1, 150) = 5.32, p = .022, η_p_^2 ^= .034), but not under a LME regression model with a random subject intercept and random slopes for noise, volatility, and their interaction (b = 0.104, z = 0.89, p = .376), suggesting that a more fine-grained measure of anxiety than a binary categorisation is required to capture individual differences in this effect.

Posterior distributions of learning parameters for low and high ANX groups are shown in Fig 8B (indecision point distributions are presented in Fig L in S1 Appendix; and comparable median split plots for Experiment 1 can be found in Fig B in S1 Appendix, although this split is based on a different median STAI-T score). Median and standard deviation values of posterior distributions of each ANX group, condition and parameter are presented in Table H in S1 Appendix. Assessing differences in ANX group-level parameters for each condition (see Table I in S1 Appendix), there was a significant ANX group difference in positive learning rate for the HVHN block, with a higher learning rate for the high versus low ANX group (HDI = [0.03, 0.20], *p*_dir_ = 97.92%, 1.03% inside the ROPE). In the low ANX group, there was a higher negative learning rate in the HVLN versus LVLN condition, i.e., a volatility-driven effect under low noise (HDI = [0.03, 0.13], *p*_dir_ = 98.95%, 0.58% inside the ROPE). In the high ANX group, there was a higher positive learning rate in HVHN than HVLN, i.e., a noise-related effect under high volatility (HDI = [0.02, 0.020], *p*_dir_ = 96.50%, 1.58% inside the ROPE), and a higher negative learning rate in HVHN compared to LVHN (HDI = [0.00, 0.12], *p*_dir_ = 95.13%, 1.92% inside the ROPE). In both groups, there was a similar higher value sensitivity in the HVLN compared to HVHN condition (low ANX: HDI = [0.19, 0.66], *p*_dir_ = 99.83%, 0.10% inside the ROPE; high ANX: HDI = [0.01, 0.53], *p*_dir_ = 95.25%, 1.78% inside the ROPE).

Regarding our original hypothesis that people higher in anxious traits would show a higher learning rate difference for increased noise (under fixed volatility) compared to less anxious individuals, we confirm here that, under high volatility, the high ANX group showed a meaningfully higher positive learning rate for high vs. low noise compared to the low ANX group (HDI = [0.00, 0.26], *p*_dir_ = 95.20%, 1.87% inside the ROPE). There were no ANX groups differences for the same comparison for the negative learning rate, nor for similar noise-related comparisons in both positive and negative learning rates when low volatility was kept fixed (all HDIs overlap 0). Overall, our RL modelling findings are different to those found in Experiment 1 in that noise- and anxiety-related differences are expressed in the positive learning rate rather than negative learning rate model parameter.

### Block order effects and attention checks

As in Experiment 1, we carried out an exploratory analysis on the effects of block order on behavioural measures, with eight block orders in total. There was only one significant but small interaction in Experiment 2, between volatility and block order on win-stay responses (F(7, 144) = 2.186, p = .039, η_p_^2 ^= .096). This appeared to be mainly driven by an interaction between the block orders also included in the original study that began with two high volatility blocks in a row (i.e., starting with either HVLN-HVHN or HVHN-HVLN): participants who had HVLN first showed more win-stay responses in high compared to low volatility, whereas those who had HVHN first showed relatively less win-stay in high compared to low volatility. We also performed an in-depth analysis on the effects of the original block orders assigned in Experiment 1 compared to the additional block orders in Experiment 2, and the effects of their interactions with volatility and noise on accuracy, win-stay and lose-shift behaviour (see Text G in S1 Appendix for full details). In summary, we found strong interactions between the original versus new block orders and volatility and noise on all three behavioural measures.

In a preregistered exploratory analysis (see https://osf.io/e2znd), we also examined how scores on intermittent attention checks were associated with changes in learning rates across volatility and noise conditions. We found indications of a relationship between attention score and changes in both positive (p = .049) and negative (p = .007) learning rate between low and high volatility conditions under low noise, i.e., HVLN vs. LVLN. See Text H and Fig M in S1 Appendix for further details and potential interpretations for these relationships.

## Discussion

Adapting to different types of uncertainty is a daily endeavour, however, few studies have explicitly teased apart the two main sources of uncertainty – stochasticity of the incoming information (noise) and the changeability of the signal over time (volatility), in both health and disorder. People with anxiety disorders are known to experience difficulties with tolerating uncertainty [2,3], making this an important avenue of research for the effective treatment of the negative effects of anxiety. Here, in two online studies, participants with a range of anxious traits attempted to learn which stimulus was optimal across time, in a fully orthogonal RL task with low or high volatility combined with low or high noise.

In both experiments, we found that participants performed better under low compared to high noise conditions, regardless of volatility level. Remarkably, even a ~ 10% difference in noise level between conditions appeared to have a greater influence on behaviour than doubling the level of volatility. There was also more win-stay and less lose-shift behaviour under low versus high noise conditions, demonstrating that the experimental manipulation of noise levels was appropriate and detected by participants. A further consistent behavioural effect across experiments was an interaction between noise and volatility on win-stay behaviour; participants exhibited more win-stay responses for high compared to low volatility under low noise, but similar or fewer win-stay responses for the same comparison under high noise. This speaks to an increased sensitivity to trial-by-trial positive outcomes when volatility levels are increased under low noise, in line with previous studies on the effects of volatility on learning rates [1,20] - however, here we find that this relationship breaks down in the context of high noise. Comparing task manipulations under a RL modelling framework, across both experiments we found higher negative learning rate and value sensitivity parameters for high compared to low volatility under low noise, with the negative learning rate finding explicitly corroborating previous studies on volatility adjustments in learning rate [1,20]. To the authors’ knowledge, few if any previous studies have demonstrated a consistent volatility-induced shift in the value sensitivity parameter. Finally, participants across both experiments demonstrated reduced value sensitivity for high compared to low noise under high volatility only, i.e., they were more exploratory in their choices in the HVHN compared to HVLN condition. Overall, through our original and replication studies, we show several consistent effects of volatility, noise and their interactions on behavioural choices and RL model parameters during learning.

In contrast, we found differing effects of anxious traits on behavioural measures and model parameters across experiments. In Experiment 1, there were more win-stay responses in general for the high compared to low ANX groups, and win-stay behaviour scaled with level of anxious traits in the full sample. There was also a greater reduction in win-stay responses and a greater increase in lose-shift responses in high versus low noise conditions for the high compared to low ANX groups. People higher in anxious traits were found to be more sensitive to differences in noise levels, with significantly less lose-shift behaviour under low compared to high noise conditions, specifically in the context of lower volatility. Finally, we found greater value sensitivity in the high compared to low ANX group for all conditions except for LVHN, demonstrating more exploitation of value differences between options in the high ANX group. In Experiment 2, there were no lose-shift interactions with anxiety. Although we also found less win-stay and more lose-shift responding in high versus low noise conditions (as in Experiment 1), this was not affected by anxious traits. Instead, there was a three-way interaction between volatility, noise and anxious traits on win-stay behaviour, with general decreases in win-stay behaviour for those higher in anxious across all conditions except for HVHN. RL modelling results provide support for this finding, showing a higher positive learning rate in the HVHN condition for the high compared to low ANX group, with no ANX group differences in the other conditions. Also in contrast to Experiment 1, there were no ANX group differences in the value sensitivity parameter. Lastly, in Experiment 1 we do not replicate previous research demonstrating that anxious people show reduced adaptability of learning rates under higher volatility [20]. In Experiment 2, although there is also no evidence for this in positive learning rate, it is indicated in negative learning rate in the context of low noise – with a significant increase in negative learning rate in the HVLN versus LVLN condition in the low ANX group, but no difference in the high ANX group.

These differences across experiments, in relation to the influence of anxious traits on how people respond to changing stochasticity and volatility, may be due to several factors. Firstly, the demographic profiles and levels of anxious symptomatology were found to be different across the two samples. Indeed, the high rate of female participants in Experiment 1 (76%) was a motivating factor in carrying out the replication study, to obtain a more balanced and representative sample. Upon comparison, participants in Experiment 1 were also younger than participants in Experiment 2, with a mean age of 24 vs. 33 years old, respectively. Participants in Experiment 1 were found to have higher levels of anxious traits than those in Experiment 2, with mean STAI-trait scores of 51 and 45, respectively. Taken together, Experiment 1 represents a younger, more anxious, female-based sample than Experiment 2. In our analysis of Experiment 2, we followed up with better-powered LME models where we could assess how both gender and age, in separate regression models, interact with anxiety levels, volatility and noise. Although we had no prior hypotheses about how gender or age would impact behavioural change under shifting noise and volatility levels, we demonstrate via multiple interactions that the combination of anxious traits, gender and/or age together play a significant role in adapting to changing uncertainty. Future studies could explicitly test this role based on pre-planned hypotheses and a suitable experimental design. Another factor that may have contributed to the difference in findings across the two experiments was the addition of four new block orders that were included in Experiment 2. These were added to establish if our findings were confined to the block orders used in Experiment 1, where the noise level changed block-to-block but the volatility level was kept fixed for two blocks in a row at a time, i.e., noise levels varied within a larger context of changing volatility. The new blocks in Experiment 2 covered the opposite scenario, with volatility level changing block-to-block and noise level fixed for two blocks at a time. In the supplement, we demonstrate the substantial extent of these differences in block order on all behavioural measures. Altogether, we believe that the combination of sample demographics, anxiety symptoms, and block order distinctions across experiments contributed to the lack of replication in Experiment 2. Given the increased sample size in Experiment 2, double that of Experiment 1, alongside a more balanced gender representation, extended block order combinations, and the inclusion of attention checks that may have motivated participants to be more alert, we emphasize the robustness of Experiment 2 in detecting meaningful and generalizable effects. The level of intricacy of these effects points towards the need for future research on learning in dynamic environments to ensure these are accounted for or fully controlled within specific target samples.

We hypothesised that learning rates should *decrease* in the high versus low noise conditions, based on RL theory [18]. This was not found to be true in either experiment, with posterior distributions of low and high noise parameters generally overlapping for a given level of volatility. Interestingly though, in both experiments we found a significant reduction in win-stay behaviour in the high compared to low noise conditions. Although previous literature has suggested that win-stay/lose-shift behaviour should map onto their respective learning rate parameters [23], our results do not support this, at least at the full sample group-level. Rather than differences in learning rate, our RL modelling results suggest that changes in noise levels may instead be represented by the value sensitivity parameter, particularly under high volatility. In both experiments, there was a clear and meaningful decrease in value sensitivity in the HVHN compared to HVLN condition, indicating more exploratory behaviour in the context of high noise. This interpretation aligns with the notion that there is less to learn from a noisier environment; but rather than reducing their learning rate to integrate feedback over a longer timescale, participants in these experiments adjusted to a more exploratory choice strategy. The extent of uncertainty in this task in general, particularly in the HVHN condition, could potentially explain why people adjust their choice strategy rather than learning rate.

Predictions from Piray and Daw [34] suggest that anxiety primarily affects inference about noise (stochasticity) but that the learner misinterprets fluctuations due to noise as a change caused by volatility. The authors present a model demonstrating greater change in a volatility parameter and a flatlined response (lesion) to stochasticity in anxious people compared to controls. Although we didn’t find a general decrease in learning rates under high vs. low noise, our original hypothesis was that if people higher in anxiety mistake an increase in noise for an increase in volatility, then we should see noise-related increases in learning rate in individuals with higher levels of anxious traits. In Experiment 1, we found more lose-shift behaviour in LVHN compared to LVLN for those with higher anxious traits. This noise-related change in lose-shift behaviour was associated with anxious traits only under low and not high volatility. Compared to the LVLN baseline condition, the high ANX group showed an increase in negative learning rate in LVHN (although not significant with these contingencies), leading to a completely overlapping distribution with the HVHN condition, whereas the low ANX group showed a significant difference between the LVHN and HVHN conditions. In Experiment 2, there was a meaningful increase in the high ANX group-level positive learning rate distribution in HVHN compared to HVLN, as well as a higher positive learning rate in the HVHN condition in the high compared to low ANX group. Taken together, although anxiety-related findings were differentially distributed across learning from either positive or negative outcomes across experiments, these findings offer some support for the predictions of Piray and Daw [34], suggesting that people with high anxiety may perceive high noise in certain contexts as being caused by an increase in volatility.

Findings from this study should be interpreted in the context of certain limitations. Firstly, our task design considered just two levels of volatility and noise; reversals occurred every 30–40 trials in low volatility and every 15–20 trials in high volatility conditions, with outcome reward contingencies of 75:25 and 65:35 for low and high noise, respectively. Future studies could include more levels of uncertainty to obtain a more fine-grained picture of how learning changes across different volatility and noise combinations and according to anxious traits. Secondly, separate hierarchical RL models were fit across the full sample, low ANX group, and high ANX group. In hierarchical models, low-level parameter estimates are pulled more closely together than if there were no higher-level distributions, a property termed “shrinkage” [36]. As is also visible from our parameter recovery simulations (Fig E in S1 Appendix), the use of individual-level parameters from these models should be interpreted with caution. In the full sample model, people with the full range of anxious traits contribute to the group-level parameters, which means that individuals with low and high anxiety are all pulled closer to the group mode. This potentially obfuscates any anxiety-related differences in individual-level parameters. Finally, in terms of task design, our experimental manipulations were in a general context of high uncertainty - a highly dynamic environment - and were not compared to stable or deterministic settings. Future studies might disentangle anxiety-related differences in stable or deterministic contexts from those involving gradual increases in volatility and/or noise.

To conclude, across two experiments we found extensive and differential effects of changing volatility and noise, and their combinations, on choice behaviour. These effects were modulated by anxious traits and sample demographics. We found the order in which participants were faced with different combinations of volatility and noise to also play a significant role in task behaviour. RL model parameters replicated previous studies showing increases in learning rate for increased volatility and furthermore hint at a greater increase in learning rate, under certain contexts, for higher environmental noise in people who have higher levels of anxious traits. Overall, our findings suggest that changes in both sources of uncertainty - stochasticity and volatility - should be carefully considered when assessing learning, in relation to anxiety and potentially other neuropsychiatric conditions.

## Materials and methods

### Ethics statement

Ethical approval for the study was granted by the Cambridge Psychology Research Ethics Committee (CPREC PRE.2019.110).

### Participants

The online Gorilla Experiment Builder platform was used to develop the RL task and host this study. Eighty participants were recruited via Prolific and were automatically redirected to Gorilla using an external URL. Inclusion criteria were that participants had to use a desktop computer (not a tablet or mobile phone), were in the age range 18–65 years, and could be from any country but had to be fluent in English. The full experiment was expected to take approximately 45 minutes to complete, for which participants were compensated £6. Participants first read through an information sheet and provided informed consent by ticking their agreement to provided statements. Participants first completed some demographic and symptoms questionnaire data, followed by the RL task.

### Experimental paradigm

Participants performed a standard probabilistic reversal learning task, choosing between a blue and orange cup on each trial. The locations of the cups were counterbalanced across trials between the left and right side of a central fixation cross. There were four blocks with a short break between blocks. The blocks were: low volatility and low noise (LVLN), low volatility and high noise (LVHN), high volatility and low noise (HVLN) and high volatility and high noise (HVHN). The study consisted of 540 trials in total, with 135 trials in each of four blocks. The average reward contingencies (noise levels) in the low and high noise conditions, regardless of volatility, were 75:35 and 65:35 respectively. Reversals in contingency occurred every 30–40 trials for the low volatility conditions and every 15–20 trials in the high volatility conditions. In Experiment 1, to ensure that results would not be due to a specific block order, half of participants completed the low volatility conditions first and the other half completed the high volatility conditions first. Low and high noise blocks were also counterbalanced within each of these groups, i.e., there were four different block orders in total. In Experiment 2, an additional four block orders were included, with participants assigned to these new block orders completing either the low or high noise conditions first, with low and high volatility counterbalanced within these groups, i.e., Experiment 2 consisted of eight block orders in total. Participants were instructed before each block to keep trying to choose the better cup, and that the better cup may change across time. The instructions highlighting how the changes might occur were as follows:

“*At any point in time one of the cups will be more likely to give you coins than the other* ***on average/over time****. Your task will be to try and work out which cup gives you more coins on average and choose that cup.*

*There is a twist though: the best cup can change – so you might initially work out that one cup gives you more coins on average than the other, but at some point without warning this will switch.*

*You’ll need to figure out when to stop choosing one cup and switch to the other. You don’t know when the cups will switch and you don’t know how often.”*

All participants were given the same sequence of trials, e.g., on a particular trial, choosing the orange cup led to the same outcome across participants. The outcome of each cup on any specific trial was always the inverse of the outcome of the other cup, i.e., if a participant chose the orange cup on trial 10 then they would have received a positive outcome (gold coin), whereas if they chose the blue cup on that trial, they would have received a negative outcome (red cross).

### Analysis *of* task behaviour

Behavioural performance was determined using measures of accuracy, win-stay responses, and lose-shift responses. In each block condition (LVLN, LVHN, HVLN, HVHN), one particular cup that was considered correct for each mini-block, i.e., between each reversal, regardless of whether there was a low or high noise associated with the reward contingency for that stimulus. Accuracy was calculated as the percentage of correct responses, collapsed across all mini-blocks per condition. Win-stay and lose-shift behaviour was assessed by calculating the number of times a participant stayed with choosing the same cup that was rewarded on the previous trial out of the total number of win outcomes on that condition (win-stay percentage), and the number of times a participant switched to choosing the other cup after receiving a loss for the original cup on the previous trial out of the total number of loss outcomes on that condition (lose-switch percentage). Since this was a learning and decision-making task with no instructions given to participants about how fast they should respond, and for which computational models were fit to choices and feedback only, reaction times were not of concern. This in line with previous studies using similar tasks [37–40]. For all analyses on task accuracy, win-stay and lose-shift behaviour, we ran repeated-measures ANOVA in JASP [41], with volatility and noise levels as within-subject factors, and in separate analyses, either anxious traits (STAI-T) as a covariate or ANX group (low/high) as a between-subjects factor. Where appropriate, these analyses were followed up by Bayesian repeated-measures ANOVA to highlight best fitting models and other models that show moderate or strong evidence, indicated by a Bayes Factor (BF) greater than 3 or 10, respectively. In line with the preregistration for Experiment 2, we also carried out logistic linear mixed-effects regression modelling on these behavioural data using the ‘lmer’ package in R. Two versions of each linear mixed-effects regression model were carried out; one that included a random intercept for subject only, and the other with a random intercept for subject in addition to random slopes for noise, volatility, and a noise * volatility interaction. The ‘bobyqa’ optimizer was used for all models with random slopes. For each regression model, the two versions were applied and compared using the likelihood ratio test. In the main text, we report the results of the more complex model only if the chi-squared test statistic *p*-value is < .05, and indicate which version is being reported.

### Reinforcement learning models

In both experiments, we compared six different hierarchical Bayesian RL models using the ‘hBayesDM’ package version 1.0.2 in R [42]. Each model was fit separately per task condition and participant group.

#### Model 1: Rescorla-Wagner (RW) two-armed bandit.

All models incorporated the Rescorla-Wagner update rule [12]; on a given trial *t*, the value of a chosen stimulus, V_c,t_, was updated based on a prediction error, i.e., the difference between the expected value of the chosen stimulus, V_c,t-1_, and the actual outcome received, O _t-1_.


$$V_{c,t}=V_{c,t-\text{1}}+\eta \left(O_{t-\text{1}}-V_{c,t-\text{1}}\right)$$ [1]

The learning rate, η(0<η<1), is a weighting on the prediction error that determines how much emphasis is placed on the current trial when updating the expected value of the stimulus.

All models also included a softmax choice function, an inverse logit transform, which relies on the difference in value between the two options to estimate the probability of choosing one stimulus over the other on a given trial.


$$p(A)=\frac{\text{1}}{\text{1}+e^{\beta (V_{B}-V_{A})}}$$ [2]

The inverse temperature parameter, β (0 < β < 10), is a weighing on the difference in value between options. We refer to the β parameter as value sensitivity, as it represents the extent to which a difference in stimulus values (obtained through an individual’s learning) determines choice, e.g., a higher β value is associated with a greater emphasis on the difference in stimulus values, whereas a low β value denotes more stochastic responses.

#### Model 2: *Reward-Punish* (*RP*).

The RP model is an expansion of the standard RW model, for which Equation 1 is split into two equations; one that is updated after receiving a reward, with a positive learning rate η_pos_, and one that is updated after receiving a punishment, with a negative learning rate η_neg_.


$$V_{c,t}=\left\{ \;\;\;\;\;\begin{array}{c} V_{c,t-\text{1}}+\eta^{rew}\left(O_{t-\text{1}}-V_{c,t-\text{1}}\right),ifO_{t-\text{1}}>\text{0}\\ V_{c,t-\text{1}}+\eta^{pun}\left(O_{t-\text{1}}-V_{c,t-\text{1}}\right),ifO_{t-\text{1}}<\text{0} \end{array}\right.$$ [3]

#### Model 3: *Experience-weighted attraction* (*EWA*).

The EWA model is used to account for a growing insensitivity to new information. In standard Rescorla-Wagner learning models, predictions are driven by the most recent experiences. With this model, which is extended in the hBayesDM package based on den Ouden et al. [39], an experience-weight parameter, called the experience decay factor ρ, denotes the importance of past experience relative to new information as the task progresses. The experience weight of the chosen stimulus on the current trial, n_c,t_, is given by:


$$n_{c,t}=n_{c,t-\text{1}}\times \rho +\text{1}$$ [4]

The value of the chosen stimulus on the current trial is then updated according to these experience weights, as well as a decay factor for previous payoffs φ, according to the following:


$$V_{c,t}=\frac{{(V}_{c,t-\text{1}}\times \varphi \times n_{c,t-\text{1}}+O_{t-\text{1}})}{n_{c,t}}$$ [5]

For ρ = 0, trial-by-trial updates are driven by the most recent experiences, but as ρ increases to 1 all trials contribute equally to the experience weight, and thus to updates in expected value of stimuli.

#### Model 4: *Fictitious Update* (*FU*).

This model not only updates values for the chosen option (V_c,t_), but also for the option that was not chosen (V_nc,t_). This is a potential strategy because of the reciprocal relationship between the options; if the outcome of choosing one option is a win, then the outcome of having chosen the other option would have been a loss. Thus, on each trial the chosen and unchosen stimuli are updated according to the following:


$$V_{c,t}=V_{c,t-\text{1}}+\eta \left(O_{t-\text{1}}-V_{c,t-\text{1}}\right)$$ [same as 1]

$$V_{nc,t}=V_{nc,t-\text{1}}+\eta \left(-O_{t-\text{1}}-V_{c,t-\text{1}}\right)$$ [6]

The negative sign in front of the outcome *O*_t-1_ captures the reciprocal relationship between options. The learning rate, η, is the same across both stimuli.

#### Model 5: Fictitious Update *+* Reward-Punish (FU-RP).

This model is a combination of both the FU and RP models, i.e., including separate reward and punishment learning rates along with value updates for both chosen and not chosen options.

#### Model 6: Fictitious Update *+* Reward-Punish *+* Indecision Point (FU-RP-IP).

This final model is the only one where an adjustment to the softmax equation (equation 2) is made, as follows:


$$p(A)=\frac{\text{1}}{\text{1}+e^{\beta (\alpha -(V_{A}-V_{B})}}$$ [7]

The indecision point, α, is the midpoint of the sigmoid function, i.e., where both options are equally likely to be selected. If this is far from 0, then there is a preference for one option over the other.

### Model fitting and validation

The hBayesDM package provides an accessible implementation of hierarchical Bayesian parameter estimation. In hierarchical models, group and individual parameter distributions are fit simultaneously, thereby mutually constraining and informing each other, resulting in greater statistical power over non-hierarchical methods [36,42–44]. Model posteriors were estimated using Markov Chain Monte Carlo (MCMC) inference. For both experiments, we fit three chains with 4,000 samples for each of the models, discarding the first 2,000 samples as burn-in. The remaining samples were used in the estimation of posterior distributions of model parameters. Convergence of the chains was confirmed using manual examination of the trace plots (hairy caterpillars, easily moving around the parameter space) and evaluation of $\hat{r}$statistics, which were all < 1.1 [45]. Model fits were compared using the leave-one-out cross-validation information criterion (LOOIC), to estimate out-of-sample prediction accuracy for each model using the log-likelihood of the posterior simulations of parameter values [46]. In each experiment, the best fitting model with the lowest LOOIC value was used for further analyses. Group-level posterior estimates of parameters were compared per task condition and participant group, and the median of individual-level parameter estimates were extracted for analyses on the relationship between learning parameters and anxious traits. Statistics on posterior distributions were carried out using the BayestestR package. For group-level comparisons, we use the 89% highest density interval (HDI), a credible interval (CI), on the difference between the posterior distributions. CIs computed with 89% intervals are deemed to be more stable than 95% intervals [36,47]. Bayesian statisticians also advise that these CIs are arbitrary and the use of, e.g., 95%, is based on frequentist statistics [47]. If the HDI doesn’t overlap zero, it implies that there is a significant difference between the posterior distributions. We also report the probability of direction (p_dir_) as an index of the existence of an effect - representing the certainty with which an effect goes in a particular direction (i.e., is positive or negative) - and the region of practical equivalence (ROPE) as an index of the significance of the effect [48]. The ROPE is the proportion of the 89% HDI of a posterior distribution that lies within a region of practical equivalence, an area around the null value enclosing values that are equivalent to the null value for practical purposes [36]. The null value is set to the -0.1 to 0.1 range of a standardized parameter, which defines a negligible effect size according to [49]. Here, we report the percentage of the whole posterior distribution contained within the ROPE. The null hypothesis was rejected if the percentage of the posterior inside the ROPE was smaller than 2.5%, i.e., the closer to zero, the better.

### Symptom measures

Levels of anxious traits were measured using the state-trait anxiety inventory (STAI) [50]. This includes 40 questions in total, with 20 covering how a person feels *right now* (state) and 20 relating to how a person feels *in general* (trait). This also uses a 4-point Likert scale, ranging from *not at all* to *very much so* responses. In this study we use anxious trait scores for further analyses, as we attempt to understand how this more stable construct is affected by noise and volatility, based on previous studies [32,35].

### Power analyses

Power analyses were carried out using the ‘pwr’ package in R. The pwr package allowed us to calculate the minimum required sample size to test a (Pearson) correlation against a constant using Fisher’s z transformation. For both experiments, we assumed to know the expected direction of the effect based on findings from the original study upon which it was based.

## Supporting information

S1 Appendix Supporting Texts, Figures and Tables. **Text A.** Experiment 1 - Power analysis. **Text B.** Experiment 1 - Power analysis of experimental design. **Text C.** Experiment 1 - Parameter Recovery. **Text D.** Experiment 2 - Power analysis. **Text E.** Experiment 2 – Effects of volatility and noise on behavioural measures. **Text F.** Experiment 2 - Including age and gender as co-variates in win-stay and lose-shift analyses. **Text G.** Experiment 2 - Effects of original vs. new block orders on behavioural measures. **Text H.** Experiment 2 – Attention checks. **Fig A.** Experiment 1 - Reaction times. **Fig B.** Experiment 1 – results when low (N = 43) and high (N = 37) ANX groups are based on a median split. **Fig C**. Experiment 1 - Alternative low and high ANX groups FU-RP models without indecision point. **Fig D.** Experiment 1 - Model validation. **Fig E.** Experiment 1 - Parameter recovery. **Fig F.** Experiment 1 - Reported FU-RP-IP model on full sample. **Fig G.** Experiment 1 - Reported FU-RP-IP model on low and high ANX groups. **Fig H.** Experiment 2 – Reaction times. **Fig I.** Experiment 2 – Gender-based interaction on win-stay behaviour. **Fig J.** Experiment 2 - Age-based interaction on lose-shift behaviour. **Fig K.** Experiment 2 – Reported FU-RP-IP model on full sample. **Fig L.** Experiment 2 - Reported FU-RP-IP model on low and high ANX groups. **Fig M.** Experiment 2 – Exploratory analysis on relationship between learning rates and a proxy measure of attention. **Table A.** Experiment 1 - Model fits of full sample: LOOIC values. **Table B.** Experiment 1 - Model fits of low and high ANX groups: LOOIC values. **Table C.** Experiment 1 - Alternative low and high ANX groups FU-RP models without indecision point. **Table D.** Experiment 1 - Median group-level parameters of reported FU-RP-IP model for the full sample. **Table E.** Experiment 1 - Median group-level parameters of reported FU-RP-IP model for low and high ANX groups. **Table F.** Experiment 2 - Median group-level parameters of reported FU-RP-IP model for the full sample. **Table G.** Experiment 2 - Behavioural results for low ANX (N = 78) and high ANX (N = 74) groups. **Table H.** Experiment 2 - Median group-level parameters of reported FU-RP-IP model for low and high ANX groups. **Table I**. Experiment 2 - Differences in ANX groups model parameter posterior distributions.
(PDF)
