# Supplementary material for: The relationship between anxious traits and learning about changes in stochasticity and volatility
Source: PLoS Comput Biol. 2025 Oct 30;21(10):e1013646. doi: 10.1371/journal.pcbi.1013646 (PMC12594353; doi:10.1371/journal.pcbi.1013646)
Supplement: S1 Appendix — Text A. Experiment 1 - Power analysis. Text B. Experiment 1 - Power analysis of experimental design. Text C. Experiment 1 - Parameter Recovery. Text D. Experiment 2 - Power analysis. Text E. Experiment 2 – Effects of volatility and noise on behavioural measures. Text F. Experiment 2 - Including age and gender as co-variates in win-stay and lose-shift analyses. Text G. Experiment 2 - Effects of original vs. new block orders on behavioural measures. Text H. Experiment 2 – Attention checks. Fig A. Experiment 1 - Reaction times. Fig B. Experiment 1 – results when low (N = 43) and high (N = 37) ANX groups are based on a median split. Fig C. Experiment 1 - Alternative low and high ANX groups FU-RP models without indecision point. Fig D. Experiment 1 - Model validation. Fig E. Experiment 1 - Parameter recovery. Fig F. Experiment 1 - Reported FU-RP-IP model on full sample. Fig G. Experiment 1 - Reported FU-RP-IP model on low and high ANX groups. Fig H. Experiment 2 – Reaction times. Fig I. Experiment 2 – Gender-based interaction on win-stay behaviour. Fig J. Experiment 2 - Age-based interaction on lose-shift behaviour. Fig K. Experiment 2 – Reported FU-RP-IP model on full sample. Fig L. Experiment 2 - Reported FU-RP-IP model on low and high ANX groups. Fig M. Experiment 2 – Exploratory analysis on relationship between learning rates and a proxy measure of attention. Table A. Experiment 1 - Model fits of full sample: LOOIC values. Table B. Experiment 1 - Model fits of low and high ANX groups: LOOIC values. Table C. Experiment 1 - Alternative low and high ANX groups FU-RP models without indecision point. Table D. Experiment 1 - Median group-level parameters of reported FU-RP-IP model for the full sample. Table E. Experiment 1 - Median group-level parameters of reported FU-RP-IP model for low and high ANX groups. Table F. Experiment 2 - Median group-level parameters of reported FU-RP-IP model for the full sample. Table G. Experiment 2 - Behavioural results for low ANX (N = 78) [file pcbi.1013646.s001.pdf]

# S1 Appendix. Supporting Texts, Figures and Tables.

# The relationship between anxious traits and learning about changes in stochasticity and volatility

Brónagh McCoy[1,2*] and Rebecca P. Lawson[1]

[1]Department of Psychology, University of Cambridge, UK

[2]Department of Forensic and Neurodevelopmental Sciences, Institute of Psychiatry, Psychology and Neuroscience, King's College London, UK

*mccoy.bronagh@gmail.com

# Supplemental Text

**Text A. Experiment 1 - Power analysis.**

We performed a power analysis to calculate a minimum sample size required to capture an expected relationship between anxious traits and a change in learning rate due to increasing volatility. This was based on the relationship between learning rate (stable – volatile) and anxious traits reported in (Browning et al., 2015). Using a reported Pearson's r-value of 0.42, we calculated that we would need a sample size of at least 34 participants to obtain .80 power with an alpha level of 0.05. Given the lack of studies testing the combined and/or interactive effect of volatility and noise, we strengthened this sample size to 80 participants.

**Text B. Experiment 1 - Power analysis of experimental design.**

We assessed the ability of experimental design to detect expected changes in win-stay and lose-shift behaviour across volatility conditions. This was implemented using a power analysis on Cohen's *d* for repeated measures (paired samples) (Lakens, 2013). Since many reinforcement learning paradigms apply a noise level similar to our low noise condition (e.g. ~75% reward contingency), we carry out this analysis on the difference between low and high volatility under low noise, i.e. HVLN – LVLN. Cohen's *d* for repeated measures ($d_{rm}$) takes the correlation between the two blocks into account. We applied equation 9 from (Lakens, 2013) to our collected data:

$$d_{rm} = \frac{\bar{x}_1 - \bar{x}_2}{SD_1^2 + SD_2^2 - 2 \times r \times SD_1 \times SD_2} \times \sqrt{2(1-r)}$$

where $\bar{x}_1/\,\bar{x}_2$ and $SD_1/\,SD_2$ are the means and standard deviations of the HVLN and LVLN blocks respectively, and *r* is the correlation between them. Applying a power calculation for a paired-samples t-test on win-stay behaviour between these blocks, with N=80 and an alpha level of 0.05, we calculated that there was 92.50% power to detect a win-stay difference between the HVLN and LVLN conditions. Using the same method, we estimated 98.77% power to detect changes in lose-shift behaviour.

**Text C. Experiment 1 - Parameter Recovery.**

Parameter recovery was performed to validate the parameter estimates and ensure that the model's parameters are identifiable (Fig E). Since the same model (FU-RP-IP) was applied to all conditions, and the general features were similar across conditions, e.g., the same number of trials and a probabilistic feedback structure with several reversals in reward contingency, here we demonstrate parameter recovery on just one of the conditions – LVLN. For each ANX group, we first generated

synthetic (simulated) datasets (100 simulations per participant) from the model using the observed (true) individual-level parameters from the fitted model. We then fit the FU-RP-IP model to the simulated data in two ways: 1) including all simulated data in one large hierarchical model per group, resulting in 2800 and 2700 fake datasets (subjects) in the low and high ANX group models, respectively, and 2) fitting separate hierarchical models per original participant, e.g., 100 fake participants per model (based on McCoy et al. 2019). This combination of analyses was inspired by Boehm et al. (2018). The medians of the estimated parameters were then compared to the known generating (original) parameters. A strong correlation between the simulated and true values is indicative of good parameter recovery.

For the first approach, using one large hierarchical model across all simulated datasets per group (see Fig E (A)), we found that individual-level parameters were, in fact, not recovered well (all $r<0.4$). Inspecting this more closely, we found extremely precise group-level posterior distributions. These simulated group-level posteriors appreciably capture the medians of the original group-level posteriors (low ANX: 0.59 (±0.07) vs. 0.60 (±0.01), 0.29 (±0.04) vs. 0.26 (±0.00), 0.58 (±0.12) vs. 0.55 (±0.01), -0.05 (±0.09) vs. -0.06 (±0.01) for true versus simulated positive learning rate, negative learning rate, value sensitivity and indecision point parameter estimates, respectively; high ANX: 0.62 (±0.06) vs. 0.61 (±0.00), 0.27 (±0.04) vs. 0.25 (±0.00), 1.00 (±0.20) vs. 0.97 (±0.02), 0.01 (±0.08) vs. -0.02 (0.01) for the same parameter comparisons). Given the large number of fake participants in the simulated models (2700 and 2800 for low and high ANX groups), we interpret the non-recoverability at the individual-level to be due to the precision of these group-level distributions. Group-level parameters are known to pull individual-level parameters more tightly together, a process called "shrinkage" (Kruschke, 2015), likely leading to the simulated and true parameters effectively being uncorrelated.

With the second approach, fitting separate hierarchical models per original participant, we found that individual-level parameters were recoverable (see Fig E (B)), with very strong correlations for negative learning rate ($r=0.965$, $p<.001$), value sensitivity ($r=0.999$, $p<.001$) and indecision point parameters ($r=0.934$, $p<.001$), and a lesser, but moderate correlation for positive learning rate ($r=0.582$, $p<.001$). Plotting the original group-level distributions beneath the simulated individual estimates, we show that the simulated individual-level positive learning rate estimates that deviate most from the true values fall within the range of the true group-level posterior distribution, i.e., in the true fitted model, the group-level estimates constrain the individual-level parameters, whereas individual-level estimates from this simulation method that only includes datasets from the same original participant, are not constrained in the same way. The relatively wider, less precise true group-level distribution for positive learning rate, compared to the other parameter distributions, is also

indicative of greater variability at the individual level, which is likely what we are capturing with the results of this simulation method.

Together, these parameter recovery methods indicate that the set of estimated parameters describes the observed data well at the individual-level and particularly well at the group-level, which is the basis for our ANX group analyses.

**Text D. Experiment 2 - Power analysis.**

We calculated a minimum sample size based on two of the main findings from the original experiment (see Fig. 3): i) the positive relationship between anxious traits and the high – low noise difference under low volatility in lose-shift behaviour, and ii) the (trending) positive relationship between anxious traits and the high – low noise difference under low volatility in negative learning rate. We estimated a sample size to meet the requirement for the weakest effect (finding (ii) above). We thus used Pearson's r-value to determine the sample size we would need to obtain .80 power with an alpha level of 0.05. Given an r of .2, we determined a sample size of 154 participants. We rounded this up to 160 to allow for even counterbalancing of block order across participants. Using the method described above, we estimated a minimum sample size of 73 participants for the stronger effect described in (i).

**Text E. Experiment 2 – Effects of volatility and noise on behavioural measures.**

Repeated-measures ANOVA were performed on the effects of volatility and noise on accuracy, win-stay, and lose shift behaviour across the full sample (N=152). There was a main effect of volatility ($F(1,151)=5.30$, $p=.023$, $\eta_p^2=.034$), noise ($F(1,151)=210.55$, $p<.001$, $\eta_p^2=0.582$) and a volatility*noise interaction effect ($F(1,151)=8.57$, $p=.004$, $\eta_p^2= .054$) on accuracy, showing higher accuracy under low volatility and low noise, with higher accuracy in low compared to high volatility under low noise, but similar accuracy under high noise across the volatility conditions. For win-stay behaviour, there was no effect of volatility, but significantly less win-stay behaviour under high compared to low noise ($F(1,151)=262.82$, $p<.001$, $\eta_p^2= .635$). There was also a volatility*noise interaction ($F(1,151)=11.17$, $p=.001$, $\eta_p^2= .069$), with more win-stay for high compared to low volatility under low noise but more win-stay for low compared to high volatility under high noise. Lastly, there was more lose-shift behaviour for high compared to low volatility ($F(1,151)=14.27$, $p<.001$, $\eta_p^2= .086$) and for high compared to low noise ($F(1,151)=80.14$, $p<.001$, $\eta_p^2= .347$), with no interaction effect ($p>.1$).

**Text F. Experiment 2 – Including age and gender as co-variates in win-stay and lose-shift analyses.**

In all of the following linear mixed effects models, gender was included as an interaction term along with volatility, noise and STAI traits. All models with a random intercept for subject and random slopes for volatility, noise and their interaction were a better fit to the data than models with a random intercept only; results of the models with random slopes are therefore reported here.

For win-stay behaviour, in addition to the main effects of noise and volatility and their interaction reported in the main text, there was also a significant interaction between volatility, anxious traits, and gender (b=-0.303, z= 2.17, p=.030) (see Fig I). Under high volatility, males with higher anxious traits showed an increase in win-stay behaviour relative to less anxious males, whereas more highly anxious females showed similar win-stay responses than their less anxious counterparts. This gender divergence for higher anxious traits was not present under low volatility. A four-way interaction between volatility, noise, anxious traits, and gender was also revealed (b=0.380, z= 2.12, p=.034), with the most noteworthy addition being for males in the high noise condition; for increasing anxiety levels, males showed an increase in win-stay behaviour in the high volatility condition, but a decrease in win-stay in the low volatility condition. In the analysis including age as a regressor, there was a trending interaction between noise, anxious traits and age only (b= -0.151, z= 1.71, p = .087). Although not significant, the direction of this trend suggests that for low noise conditions, there was little effect of age on win-stay for people higher in anxiety, but for less anxious people, the older people were the more win-stay behaviour they displayed under these more stable conditions.

Assessing lose-shift behaviour, we found an interaction between gender and noise only (b= -0.188, z=2.78, p=.005) with similar lose-shift responses across gender under low noise, but more lose-shift behaviour in females compared to males under high noise. A model including age as a regressor revealed a significant interaction between noise, anxious traits and age (b=0.076, z= 2.09, p=.036), revealing differential age effects across low and high noise; under high noise, there were similar profiles of decreasing lose-shift behaviour for increasing age regardless of anxious traits, but under

low noise, there was a fully interactive effect of age and anxiety, with older participants showing more lose-shift responses than younger participants at low anxiety levels, whereas at higher anxiety levels, older participants exhibited fewer lose-shift responses than younger participants (see S10 Fig).

**Text G. Experiment 2 - Effects of original vs. new block orders on behavioural measures.**

In Experiment 1, there were four different block orders across participants (i.e., N=80, leading to 20 participants per block order). All block orders consisted of two consecutive blocks of the same volatility level, with different combinations of noise applied within this structure. Here is an example of a block order given to a participant: 1. LVLN, 2. LVHN, 3. HVLN, 4. HVHN, and for another participant: 1. HVHN, 2. HVLN, 3. LVHN, 4. LVLN. In Experiment 2, we added four new block orders, leading to eight in total (again 20 participants per block order, given the larger sample size of N=160). The new block orders consisted of two consecutive blocks of the same noise level, with different combinations of volatility applied e.g., for one participant: 1. LVLN, 2. HVLN, 3. LVHN, 4. HVHN, and for another: 1. HVHN, 2. LVHN, 3. HVLN, 4. LVLN. Taking data from Experiment 2, we assessed for any effects of the original block order (always with two blocks of the same volatility level in a row) compared to the new block orders (always with two blocks of the same noise level in a row). We carried out logistic mixed effects modelling on trial-by-trial accuracy, win-stay and lose-shift behaviour, including an "original block orders" flag (original=0, new=1) as a dependent variable, along with volatility (low=0, high=1) and noise level (low=0, high=1). Here we report only the main or interactive effects of this "original block orders" flag. Results of all linear mixed effects models below include subject as a random intercept and volatility, noise and their interaction as random slopes.

*Accuracy*

Examining task accuracy, there were very strong effects of block order, including – a positive main effect ($b=0.483$, $z=5.32$, $p<.001$), i.e., those with the new block orders were more accurate; a negative volatility by block order interaction ($b=-0.576$, $z=7.27$, $p<.001$), with fewer correct responses for original compared to new block orders under low volatility but more comparable accuracy under high volatility; and also a negative noise by block order interaction ($b=-0.506$, $z=8.59$, $p<.001$), again with

worse performance for original vs. new block orders under low noise but similar performance under high noise. There was also a full positive interaction between volatility, noise and block order (b=0.566, z=6.62, p<.001), showing similar (low) accuracy in high noise conditions regardless of volatility level or block order, but opposing effects of block order on accuracy under low noise across volatility levels. Under low noise and low volatility, people assigned to the new block orders performed similarly to under high noise, but people given the original block orders were much more accurate. However, under low noise and high volatility, people performed similarly across the original and new block orders.

*Win-stay and Lose-Shift Behaviour*

There was a significant negative interaction between volatility and block orders flag (b=-0.390, z= 2.64, p=.008) and also between noise and block orders flag (b=-0.365, z=3.41, p<.001) on win-stay behaviour. People undergoing the new block orders showed slightly more win-stay responses under low volatility than original block orders, but under high volatility, those assigned the original block orders show more win-stay responses. Under low noise, people show similar win-stay behaviour regardless of block orders, but for high noise, people with the original block orders show fewer win-stay responses. There were no significant interactions between block order and noise or volatility on lose-shift behaviour.

**Text H. Experiment 2 – Attention checks.**

In a preregistered exploratory analysis, we examined how scores on intermittent attention checks were related to changes in learning rates across volatility and noise conditions. These checks occurred twice per condition (135 trials), i.e., were relatively sparse; participants were asked to provide a binarized response to the question "did you just receive a coin?" (yes/no) or "which cup did you just choose?" (orange/blue), with a maximum score of eight across the task. We found that people who were more attentive to, or kept in mind, choices and outcomes from the just-completed trial, i.e., who scored higher on the proxy measure, showed a reduced or blunted increase in positive learning rate (r =-0.161, p =.049) and negative learning rate (r=-0.22, p=.007) in the high compared to low volatility condition under low noise, i.e., HVLN – LVLN (see Fig M). Note that this is the opposite direction to

the full sample group-level differences in both Experiments (Fig 4B and Fig 8B), which show an increase in positive (Experiment 1) and negative learning rates (Experiment 1 and 2) for high compared to low volatility in the context of low noise.

In summary, we found that those who paid more attention to trial-by-trial outcomes, as captured by a proxy measure of attention, showed reduced learning rate in HVLN, and a diminished HVLN – LVLN difference in both positive and negative learning rate compared to those who paid less attention. This is an unexpected finding; RL theory posits that when environmental volatility goes up, people should heighten their sensitivity to trial-by-trial outcomes and prediction errors (i.e., increase their learning rate), to increase the chance of noticing environmental change as it occurs (Yu et al., 2005). This finding could be interpreted in several ways; firstly, those who were paying more attention, according to our measure, may have been holding the previous trial's information in working memory for longer, as they tried to integrate it with previous trials. This type of integration over previous trials coincides with a reduced sensitivity to individual trial outcomes, resulting in a lower learning rate. Increased attention, therefore, may not necessarily indicate an increased sensitivity to trial-by-trial outcomes. Another interpretation may be that participants who were paying strong attention to the task were aware of the extent of its changeability and opted for a more cautious strategy across the task to account for how much information they could truly garner from trial to trial. Interestingly, this lowered adaptability of learning rate to a change in volatility for those with a higher attention score is reminiscent of previous studies demonstrating that anxious people show reduced volatility-driven adaptability of learning rate (Browning et al., 2015; Huang et al., 2017). Future research could investigate the effects of attention and/or working memory on learning rate changes in response to fluctuating volatility during learning.

# Supplemental Figures

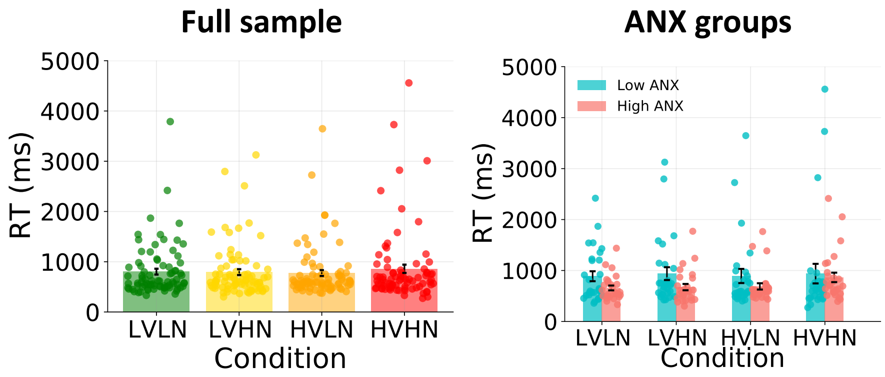


**Fig A. Experiment 1 - Reaction times.** Repeated-measures ANOVA on RTs showed no main or interactive effects of volatility or noise, and no interaction between ANX groups and either of these conditions (all p>.1).

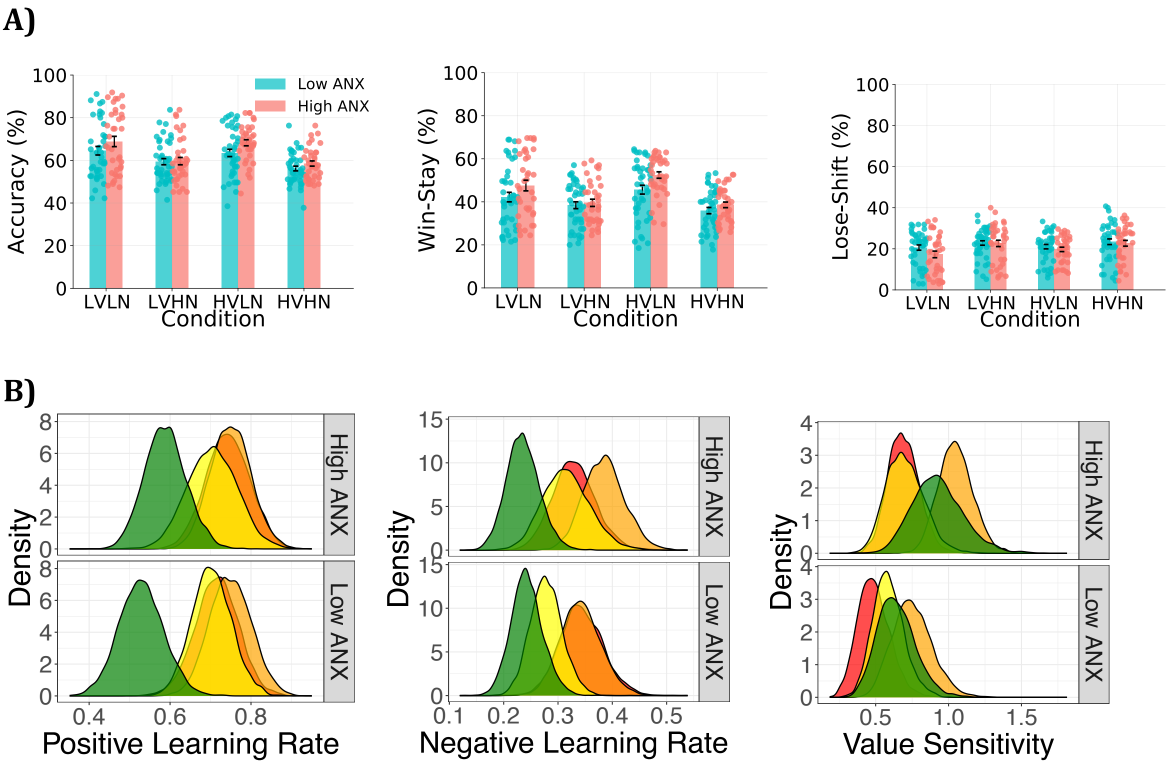


**Fig B. Experiment 1 – results when low (N = 43) and high (N= 37) ANX groups are based on a median split.** There were 7 participants with exactly the median STAI-T cut-off score of 50 – here they have been allocated to the low ANX group. **A)** *Behavioural results*. Repeated-measures ANOVA showed no interaction between volatility and ANX group for any measure, but a significant noise x ANX group interaction on win-stay behaviour (F(1,78)=7.095, p=.009, $\eta_p^2$=.083), and a trending noise x ANX group interaction on lose-shift behaviour (F(1,78)=3.095, p=.082, $\eta_p^2$=.038). These interactions followed the same pattern as reported in the main analysis. **B)** *Reinforcement learning group-level results*. The high ANX group showed higher value sensitivity than low ANX in the HVLN condition only (HDI=[0.01,0.58], $p_{dir}$=94.47%, 2.15% inside the ROPE), with HDIs slightly overlapping zero for the LVLN and HVHN conditions. All other within-group

comparisons reflect those report in the main analysis, except for the HVHN – LVHN difference in negative learning rate in the low ANX group, which was not a meaningful difference here (HDI=[-0.02, 0.14], $p_{dir}$=91.02%, 2.83% inside the ROPE). This may be due to the greater number of people with higher ANX traits now included relative to the main analysis.

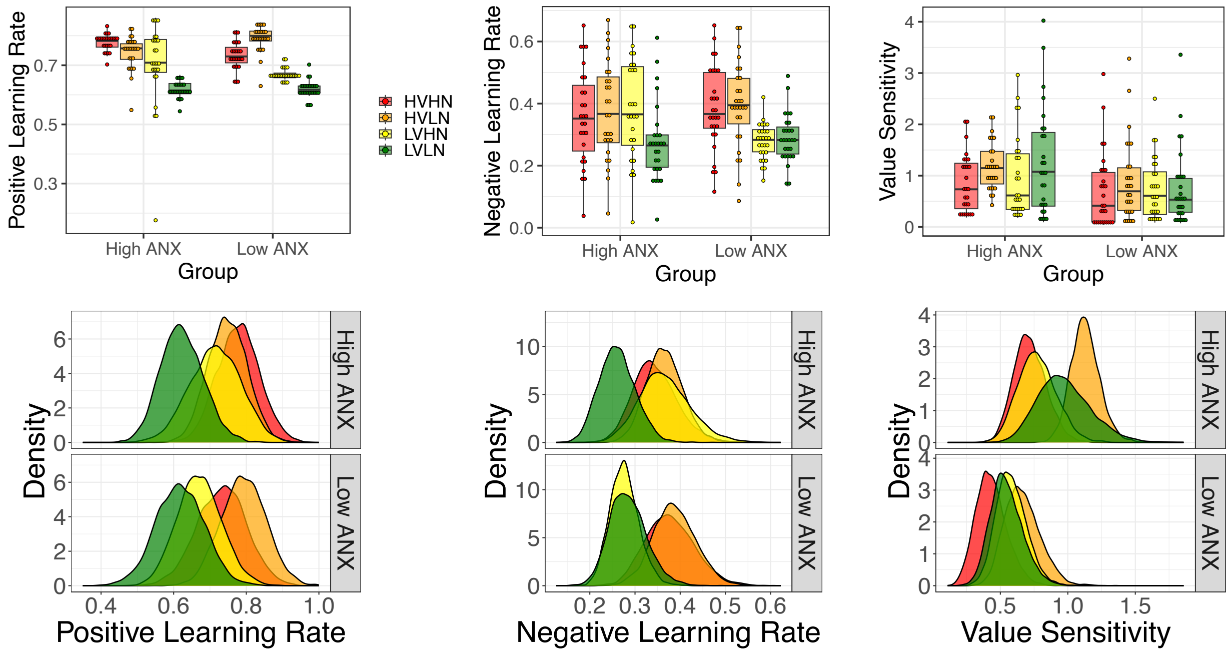


**Fig C. Experiment 1 - Alternative low and high ANX groups FU-RP models without indecision point.** Individual-level parameter estimates and group-level distributions for positive and negative learning rates and value sensitivity parameters.

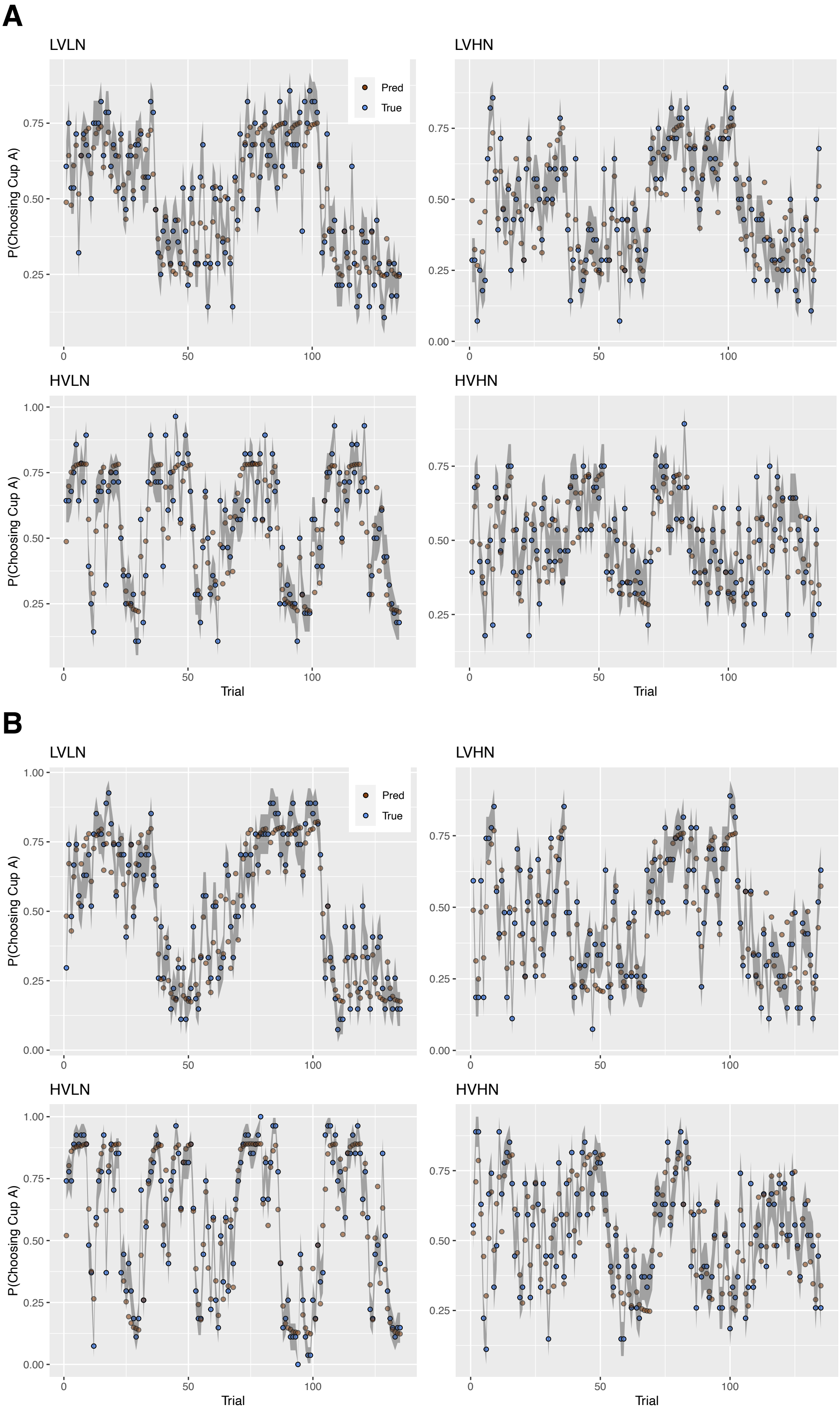


**Fig D. Experiment 1 - Model validation.** Posterior predictive checks (PPCs) on reported hBayesDM models for **(A)** the low ANX group, and **(B)** the high ANX group. Predicted choices were found to largely track true choices across time, for each condition in each ANX group. Error bars represent 95% CIs around the mean.

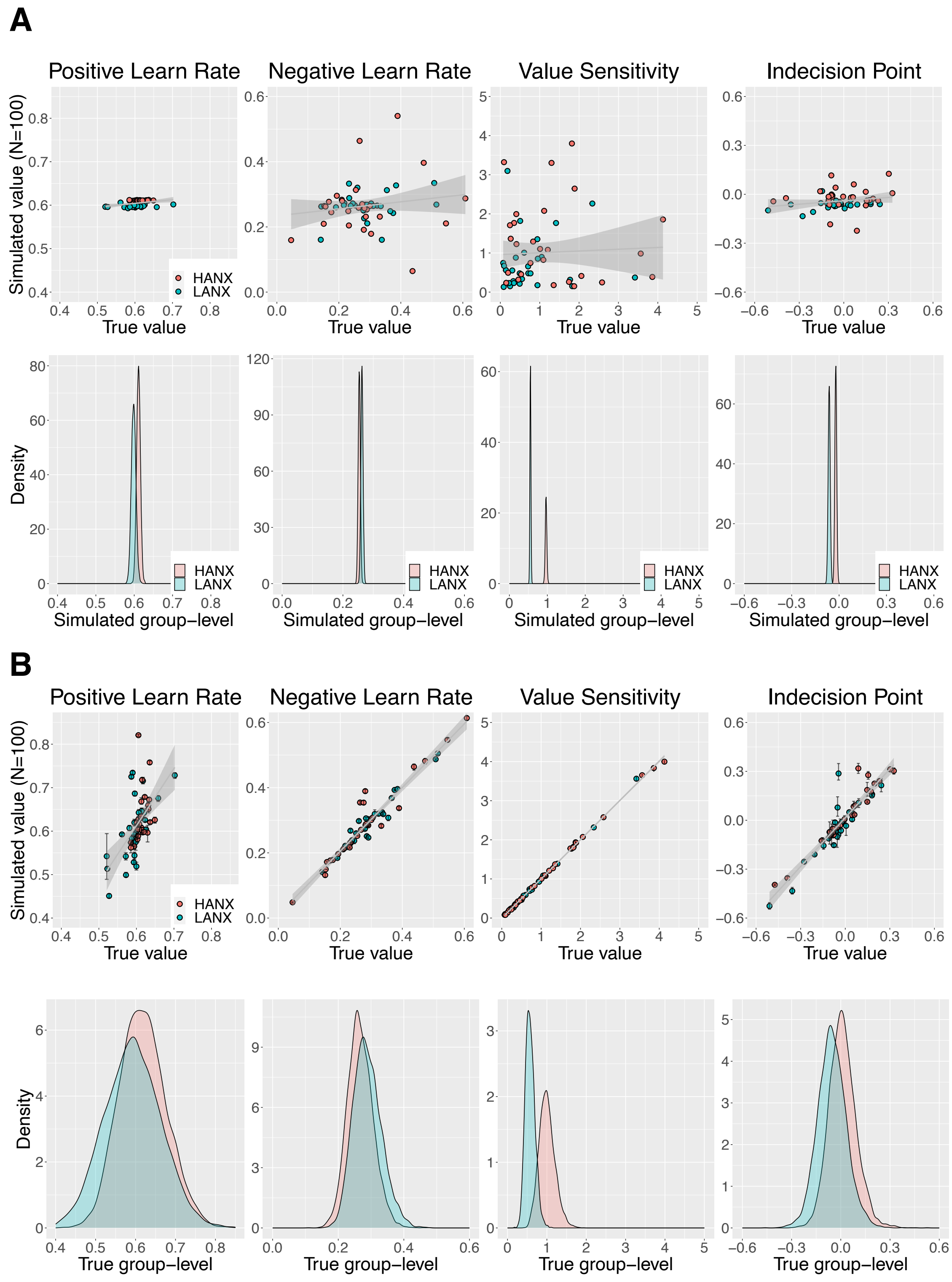


**Fig E. Experiment 1 – Parameter recovery**. **A)** Simulation method 1: 100 synthetic datasets were generated per original participant and included in one large hierarchical model per low and high ANX group. Individual-level estimates were not recoverable, i.e., true and simulated values were not strongly correlated (upper panel). Group-level distributions were recoverable, however, and highly precise (lower panel). **B)** Simulation method 2: 100 simulated datasets were fit in a single hierarchical model per original participant (based on McCoy et al. 2019). True and simulated individual-level parameters were highly correlated (r>0.9), except for a moderately-correlated positive learning rate (r=0.582) (upper panel). The true group-level posteriors (lower panel) show a relatively wider distribution for positive learning rate, likely constraining more variable individual-level estimates. Note that the same x-axis range is used for all plots of the same parameter for comparison purposes. Individual-level error bars represent ±1 SD around the mean of the simulated estimates.

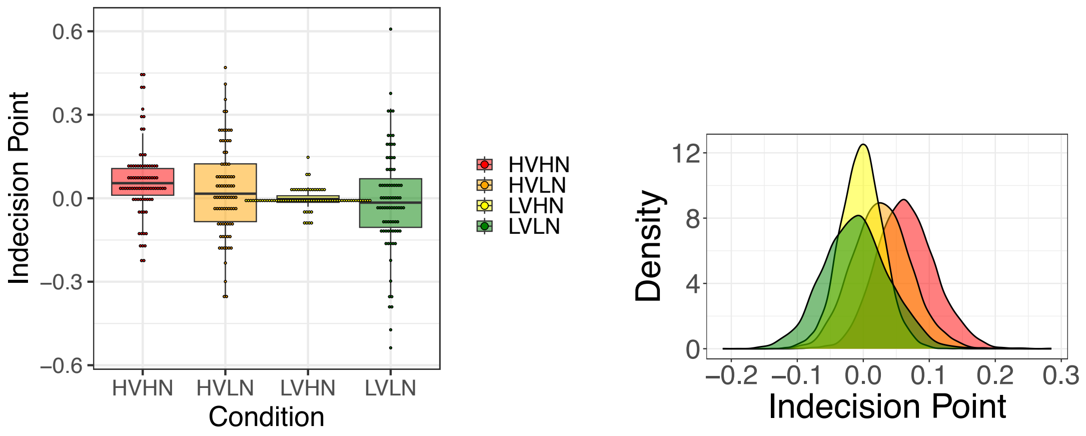


**Fig F. Experiment 1 - Reported FU-RP-IP model on full sample.** Individual-level parameter estimates (left) and group-level distributions (right) are shown for the indecision point parameter.

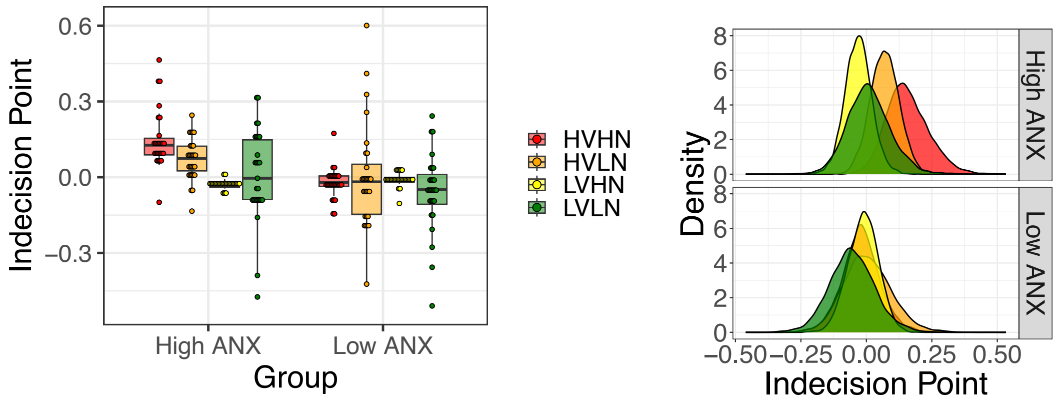


**Fig G. Experiment 1 - Reported FU-RP-IP model on low and high ANX groups.** Individual-level parameter estimates (left) and group-level distributions (right) are shown for the indecision point parameter.

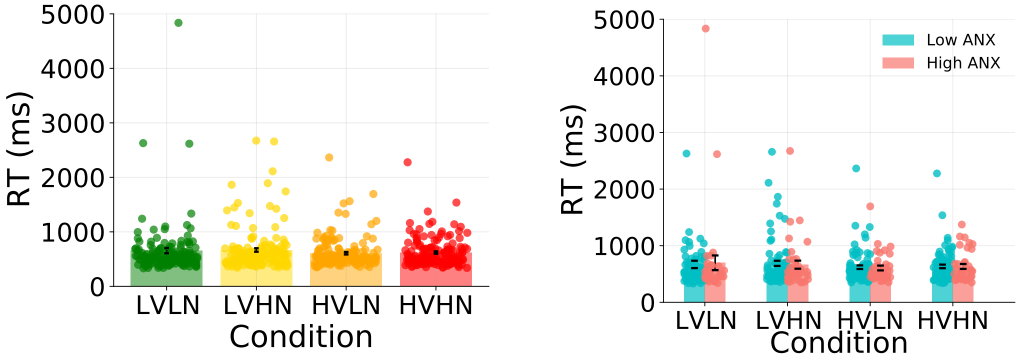


**Fig H. Experiment 2 – Reaction times.** Repeated-measures ANOVA on RTs showed no main or interactive effects of volatility or noise, and no interaction between ANX groups and either of these conditions (all p>.05).

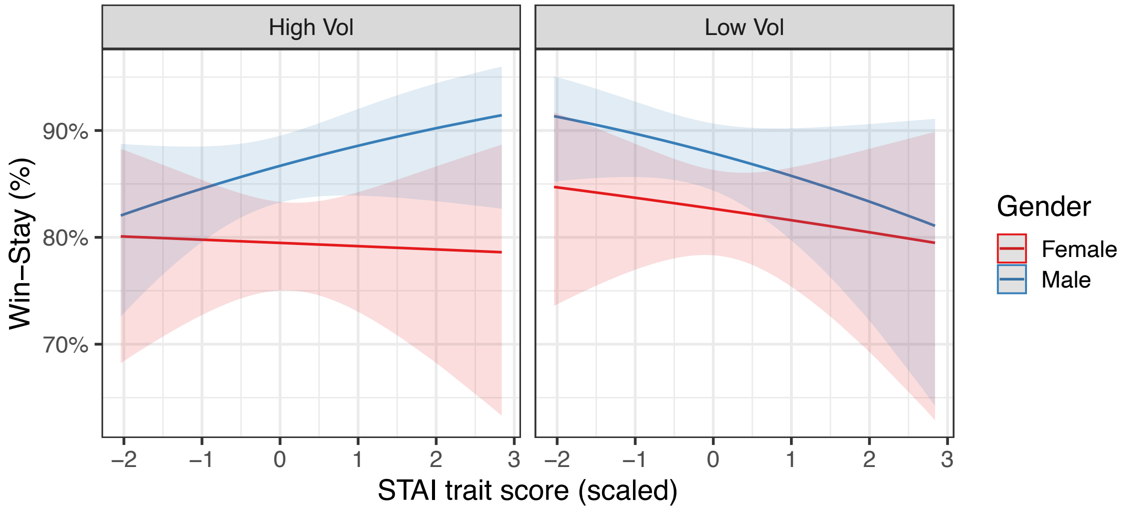


**Fig I. Experiment 2 – Gender-based interaction on win-stay behaviour.** Interaction between volatility, anxious traits, and gender (p=.030). Under high volatility, males with higher anxious traits showed an increase in win-stay behaviour relative to less anxious males, whereas more highly anxious females showed similar win-stay responses than females with fewer anxious traits. For low volatility, there were also fewer win-stay responses with increasing anxious traits, but the gender-based gap was reduced at higher anxiety levels.

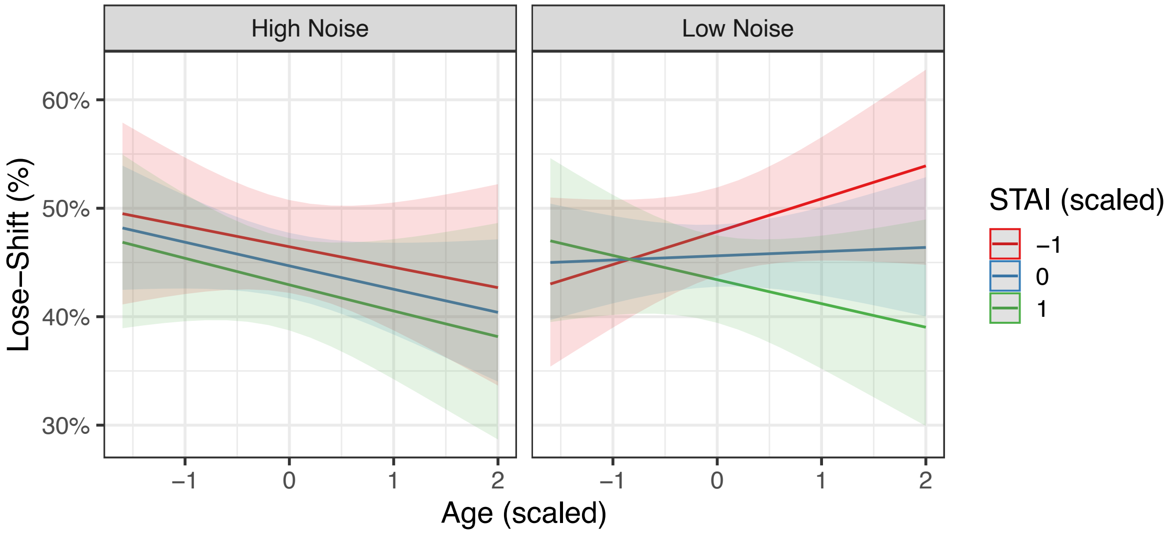


**Fig J. Experiment 2 – Age-based interaction on lose-shift behaviour.** Interaction between noise, anxious traits and age (p=.036). In the high noise conditions, participants showed a decrease in lose-shift behaviour for increasing age regardless of anxiety levels. Under low noise, there was a fully interactive effect of age and anxiety; older participants showed more lose-shift behaviour than younger participants at low anxiety levels, whereas at higher anxiety levels, older participants exhibited fewer lose-shift responses than younger participants.

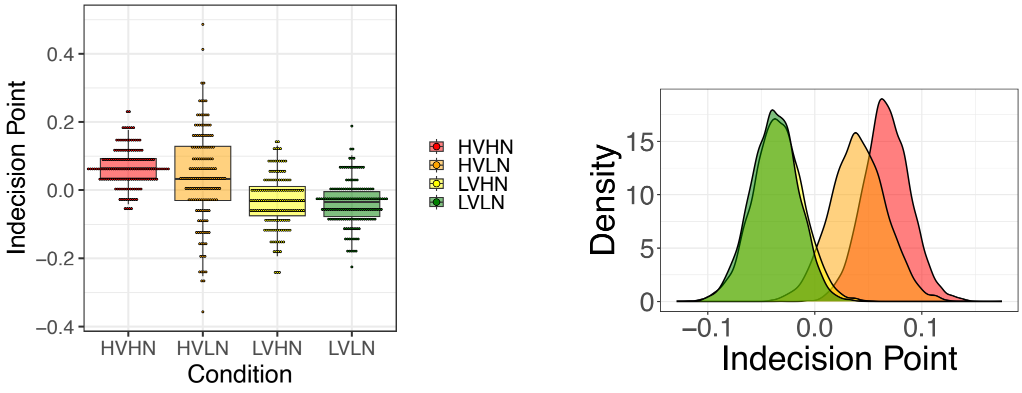


**Fig K. Experiment 2 – Reported FU-RP-IP model on full sample.** Individual-level parameter estimates (left) and group-level distributions (right) for the indecision point parameter.

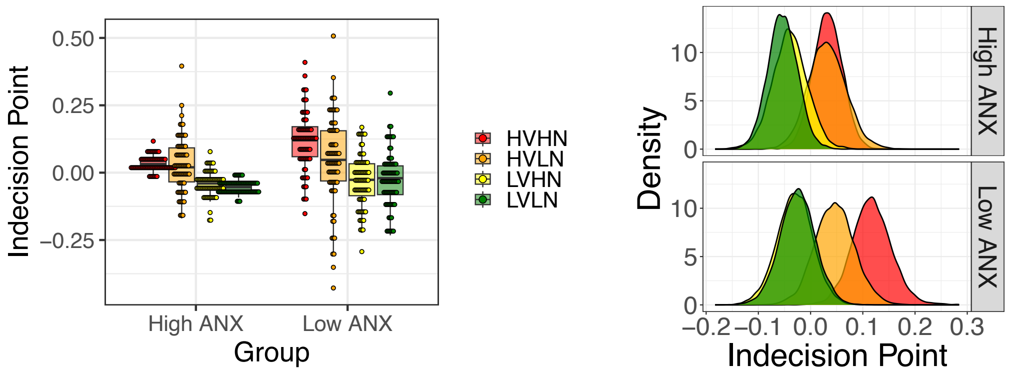


**Fig L. Experiment 2 - Reported FU-RP-IP model on low and high ANX groups.** Individual-level parameter estimates (left) and group-level distributions (right) for the indecision point parameter.

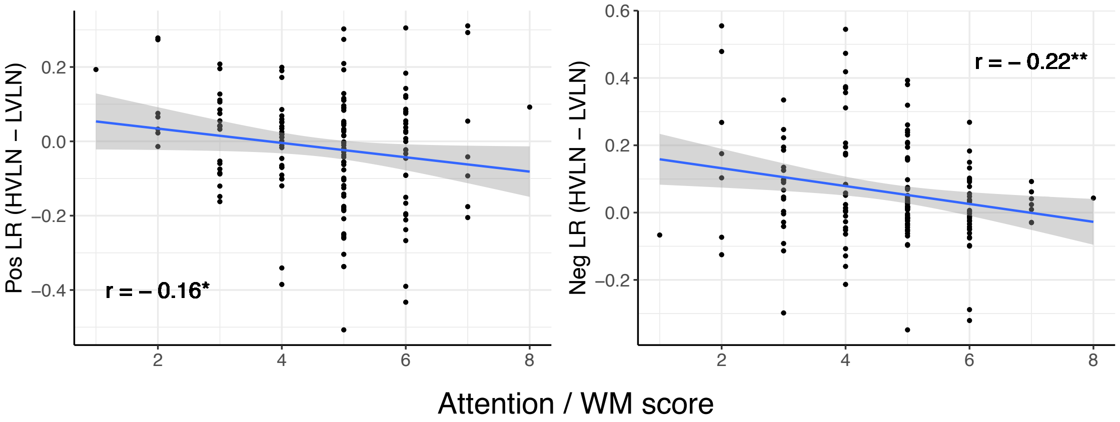


**Fig M. Experiment 2 – Exploratory analysis on relationship between learning rates and a proxy measure of attention.** Participants who were more attentive to choices and outcomes from the just-completed trial, i.e., who scored higher on the proxy attention measure, showed a reduced or blunted increase in positive learning rate (p =.049) and negative learning rate (p=.007) in the HVLN compared to LVLN condition.

# Supplemental Tables

**Table A. Experiment 1 - Model fits of full sample: LOOIC values.** The best fitting model was the fictitious update model with both reward and punishment learning rates and indecision point parameters (FU-RP-IP). In fictitious update models, the expected value of the non-chosen stimulus also gets updated on each trial, based on the feedback received for the chosen stimulus. A similar model without the indecision point (FU-IP) was the best fit for the LVHN condition, although LOOIC values are close between the two models.

| | ***Full sample*** | | | |
|---|---|---|---|---|
| ***Model name*** | LVLN | LVHN | HVLN | HVHN |
| RW | 11385.90 | 12421.94 | 10691.67 | 12633.57 |
| RP | 10994.43 | 12258.45 | 10607.50 | 12407.38 |
| EWA | 11330.21 | 12354.92 | 10674.17 | 12525.74 |
| FU | 11166.43 | 12580.26 | 10854.55 | 12767.17 |
| FU - RP | 10996.56 | **12032.90** | 10555.06 | 12216.62 |
| **FU - RP - IP** | **10962.73** | 12036.18 | **10530.48** | **10530.48** |

**Table B. Experiment 1 - Model fits of low and high ANX groups: LOOIC values.** The best fitting model was FU-RP-IP. The FU-IP model was best for the LVHN condition in the low ANX group only, although LOOIC values were only slightly higher for the FU-RP-IP model.

| ***Model name (low ANX)*** | LVLN | LVHN | HVLN | HVHN |
|---|---|---|---|---|
| RW | 4316.08 | 4541.59 | 4017.54 | 4584.80 |
| RP | 4318.65 | 4502.56 | 4025.04 | 4562.70 |
| EWA | 4309.62 | 4536.39 | 4005.97 | 4578.07 |
| FU | 4338.68 | 4611.30 | 4095.84 | 4677.84 |
| FU - RP | 4306.94 | **4423.33** | 4018.01 | 4474.27 |
| **FU - RP - IP** | **4304.15** | 4428.84 | **4005.55** | **4473.99** |

| ***Model name (high ANX)*** | LVLN | LVHN | HVLN | HVHN |
|---|---|---|---|---|
| RW | 3579.03 | 4103.30 | 3360.10 | 4273.83 |
| RP | 3390.29 | 4079.43 | 3322.79 | 4129.09 |
| EWA | 3574.57 | 4090.30 | 3357.82 | 4170.01 |
| FU | 3472.94 | 4169.32 | 3421.51 | 4219.18 |
| FU - RP | 3399.31 | 3970.87 | 3297.38 | 4035.25 |
| **FU - RP - IP** | **3392.00** | **3967.30** | **3297.03** | **4033.79** |

**Table C. Experiment 1 - Alternative low and high ANX groups FU-RP models without indecision point.** Differences in model parameter distributions between low and high ANX groups.

| | Between-group HDI: High – Low ANX | | | |
|---|---|---|---|---|
| **Model parameter** | LVLN | LVHN | HVLN | HVHN |
| Pos. learning rate | -0.14 0.14 | -0.11 0.19 | -0.18 0.08 | -0.10 0.19 |
| Neg. learning rate | -0.12 0.07 | -0.01 0.19 | -0.12 0.08 | -0.15 0.08 |
| Value sensitivity | **0.08 0.80*** | -0.10 0.48 | **0.18 0.72*** | **0.04 0.57*** |

**Table D. Experiment 1 - Median group-level parameters of reported FU-RP-IP model for the full sample.**

| Model parameter | *Full sample* | | | |
|---|---|---|---|---|
| | LVLN | LVHN | HVLN | HVHN |
| | *M (SD)* | *M (SD)* | *M (SD)* | *M (SD)* |
| Pos. learning rate | 0.56 (0.04) | 0.71 (0.04) | 0.76 (0.04) | 0.73 (0.04) |
| Neg. learning rate | 0.24 (0.02) | 0.29 (0.03) | 0.36 (0.03) | 0.34 (0.03) |
| Value sensitivity | 0.74 (0.11) | 0.62 (0.08) | 0.88 (0.10) | 0.57 (0.08) |
| Indecision Point | -0.01 (0.05) | 0.00 (0.03) | 0.03 (0.04) | 0.07 (0.04) |

**Table E. Experiment 1 - Median group-level parameters of reported FU-RP-IP model for low and high ANX groups.**

| Model parameter | *Low ANX* | | | |
|---|---|---|---|---|
| | LVLN | LVHN | HVLN | HVHN |
| | *M (SD)* | *M (SD)* | *M (SD)* | *M (SD)* |
| Pos. learning rate | 0.59 (0.07) | 0.68 (0.06) | 0.77 (0.07) | 0.74 (0.07) |
| Neg. learning rate | 0.29 (0.04) | 0.28 (0.03) | 0.39 (0.05) | 0.39 (0.06) |
| Value sensitivity | 0.58 (0.12) | 0.58 (0.12) | 0.68 (0.14) | 0.42 (0.11) |
| Indecision Point | -0.05 (0.09) | -0.01 (0.06) | 0.00 (0.09) | -0.02 (0.07) |

| Model parameter | *High ANX* | | | |
|---|---|---|---|---|
| | LVLN | LVHN | HVLN | HVHN |
| | *M (SD)* | *M (SD)* | *M (SD)* | *M (SD)* |
| Pos. learning rate | 0.62 (0.06) | 0.72 (0.07) | 0.76 (0.05) | 0.80 (0.06) |
| Neg. learning rate | 0.27 (0.04) | 0.37 (0.06) | 0.37 (0.04) | 0.35 (0.05) |
| Value sensitivity | 1.00 (0.20) | 0.76 (0.14) | 1.12 (0.11) | 0.72 (0.11) |
| Indecision Point | 0.01 (0.08) | -0.03 (0.05) | 0.07 (0.06) | 0.16 (0.08) |

**Table F. Experiment 2 - Median group-level parameters of reported FU-RP-IP model for the full sample.**

| Model parameter | *Full sample* | | | |
|---|---|---|---|---|
| | LVLN | LVHN | HVLN | HVHN |
| | *M (SD)* | *M (SD)* | *M (SD)* | *M (SD)* |
| Pos. learning rate | 0.74 (0.03) | 0.79 (0.03) | 0.74 (0.03) | 0.78 (0.03) |
| Neg. learning rate | 0.26 (0.02) | 0.29 (0.02) | 0.32 (0.02) | 0.32 (0.02) |
| Value sensitivity | 0.95 (0.08) | 0.87 (0.08) | 1.17 (0.09) | 0.82 (0.07) |
| Indecision Point | -0.04 (0.02) | -0.03 (0.02) | 0.04 (0.03) | 0.07 (0.02) |

**Table G. Experiment 2 - Behavioural results for low ANX (N=78) and high ANX (N=74) groups.** Average accuracy, reaction time (RT), win-stay, and lose-shift behaviour is shown per condition. Differences in behavioural measures for low vs. high noise and low vs. high volatility are also displayed. Numbers in brackets represent ±1 SD.

| | *Low ANX* | | | | | |
|---|---|---|---|---|---|---|
| ***Measure*** | LVLN | LVHN | HVLN | HVHN | Low - High Noise | Low - High Vol |
| Accuracy (%) | 70.70 (13.68) | 60.06 (8.59) | 67.96 (10.12) | 58.94 (7.71) | 9.67 (8.60) | 1.92 (7.83) |
| RT (ms) | 673 (593) | 690 (415) | 620 (293) | 631 (290) | -14 (284) | 56 (355) |
| Win-stay (%) | 50.24 (14.18) | 42.20 (9.04) | 52.92 (11.67) | 39.19 (8.23) | 10.88 (8.81) | 0.17 (7.15) |
| Lose-shift (%) | 17.90 (9.41) | 21.95 (9.91) | 19.22 (7.55) | 23.68 (9.36) | -4.25 (5.88) | -1.52 (6.17) |

| | *High ANX* | | | | | |
|---|---|---|---|---|---|---|
| ***Measure*** | LVLN | LVHN | HVLN | HVHN | Low - High Noise | Low - High Vol |
| Accuracy (%) | 70.37 (13.97) | 58.79 (11.00) | 66.92 (9.73) | 60.57 (9.87) | 9.09 (7.34) | 0.58 (6.74) |
| RT (ms) | 638 (574) | 643 (363) | 595 (260) | 609 (225) | -10 (367) | 38 (293) |
| Win-stay (%) | 50.68 (14.36) | 41.66 (11.24) | 50.54 (11.81) | 40.49 (9.67) | 9.53 (6.50) | 0.66 (5.99) |
| Lose-shift (%) | 16.14 (9.08) | 19.92 (9.27) | 18.38 (7.16) | 21.78 (9.52) | -3.59 (4.89) | -2.05 (5.45) |

**Table H. Experiment 2 - Median group-level parameters of reported FU-RP-IP model for low and high ANX groups.**

| | *Low ANX* | | | |
|---|---|---|---|---|
| Model parameter | LVLN | LVHN | HVLN | HVHN |
| | *M (SD)* | *M (SD)* | *M (SD)* | *M (SD)* |
| Pos. learning rate | 0.73 (0.04) | 0.75 (0.04) | 0.73 (0.04) | 0.70 (0.04) |
| Neg. learning rate | 0.27 (0.02) | 0.31 (0.03) | 0.35 (0.02) | 0.31 (0.03) |
| Value sensitivity | 1.01 (0.11) | 0.97 (0.10) | 1.27 (0.12) | 0.84 (0.09) |
| Indecision Point | -0.02 (0.03) | -0.03 (0.03) | 0.05 (0.04) | 0.12 (0.04) |

| | *High ANX* | | | |
|---|---|---|---|---|
| Model parameter | LVLN | LVHN | HVLN | HVHN |
| | *M (SD)* | *M (SD)* | *M (SD)* | *M (SD)* |
| Pos. learning rate | 0.73 (0.04) | 0.80 (0.04) | 0.71 (0.04) | 0.82 (0.04) |
| Neg. learning rate | 0.26 (0.02) | 0.26 (0.03) | 0.29 (0.03) | 0.33 (0.03) |
| Value sensitivity | 0.91 (0.11) | 0.77 (0.11) | 1.09 (0.12) | 0.82 (0.11) |
| Indecision Point | -0.05 (0.03) | -0.04 (0.03) | 0.03 (0.04) | 0.03 (0.03) |

**Table I. Experiment 2 - Differences in ANX groups model parameter posterior distributions**. Highest density intervals (HDI) on group and condition parameter posterior distribution differences, showing the lower and upper bounds of the 89% HDI. HDI that does not overlap 0 indicates a meaningful difference between distributions. Between-group HDI per condition (top), and between-condition HDI for each group (middle: Low ANX, bottom: High ANX).

| | Between-group HDI: High – Low ANX | | | |
|---|---|---|---|---|
| **Model parameter** | LVLN | LVHN | HVLN | HVHN |
| Pos. learning rate | -0.08 0.09 | -0.04 0.14 | -0.11 0.08 | **0.03 0.20*** |
| Neg. learning rate | -0.06 0.04 | -0.11 0.01 | -0.11 0.00§ | -0.05 0.08 |
| Value sensitivity | -0.36 0.15 | -0.42 0.05 | -0.45 0.09 | -0.25 0.21 |
| Indecision point | -0.10 0.04 | -0.09 0.07 | -0.10 0.07 | **-0.16 -0.01*** |

| **Model parameter** | Between-condition HDI: Low ANX | | | | |
|---|---|---|---|---|---|
| | LVLN - LVHN | HVLN - LVLN | HVHN - LVHN | HVLN - HVHN | LVLN - HVHN |
| Pos. learning rate | -0.11 0.07 | -0.08 0.09 | -0.14 0.04 | -0.06 0.13 | -0.07 0.11 |
| Neg. learning rate | -0.11 0.02 | **0.03 0.13*** | -0.07 0.06 | -0.03 0.10 | -0.10 0.02 |
| Value sensitivity | -0.20 0.27 | -0.00 0.52§ | -0.33 0.10 | **0.19 0.66*** | -0.04 0.40 |
| Indecision point | -0.09 0.11 | -0.01 0.16 | **0.07 0.23*** | -0.15 0.02 | **-0.22 -0.06*** |

| **Model parameter** | Between-condition HDI: High ANX | | | | |
|---|---|---|---|---|---|
| | LVLN – LVHN | HVLN – LVLN | HVHN – LVHN | HVLN – HVHN | LVLN – HVHN |
| Pos. learning rate | -0.15 0.02 | -0.11 0.07 | -0.06 0.11 | **-0.20 -0.02*** | **-0.17 -0.00*** |
| Neg. learning rate | -0.08 0.06 | -0.02 0.09 | **0.00 0.12*** | -0.10 0.03 | **-0.13 -0.01*** |
| Value sensitivity | -0.17 0.44 | -0.09 0.44 | -0.19 0.31 | **0.01 0.53*** | -0.16 0.34 |
| Indecision point | -0.10 0.07 | **0.01 0.16*** | -0.00 0.14§ | -0.07 0.08 | **-0.15 -0.02*** |

## Supplemental References


Browning M, Behrens TE, Jocham G, O'Reilly JX, Bishop SJ. Anxious individuals have difficulty learning the causal statistics of aversive environments. Nat Neurosci. 2015;18(4):590-596.

Boehm U, Marsman M, Matzke D, Wagenmakers EJ. On the importance of avoiding shortcuts in applying cognitive models to hierarchical data. Behavior Research Methods. 2018;50:1614–1631.

Huang H, Thompson W, Paulus MP. Computational dysfunctions in anxiety: failure to differentiate signal from noise. Biol Psychiatry. 2017;82(6):440–446.

Kruschke JK. Doing bayesian data analysis: A tutorial with r, JAGS, and stan. 2nd ed. Academic Press; 2015.

Lakens D. Calculating and reporting effect sizes to facilitate cumulative science: a practical primer for t-tests and ANOVAs. Frontiers in Psychology. 2013; 4(863).

McCoy B, Jahfari S, Engels G, Knapen T, Theeuwes J. Dopaminergic medication reduces striatal sensitivity to negative outcomes in Parkinson's disease. Brain. 2019;142(11):3605–3620.

Yu AJ, Dayan P. Uncertainty, neuromodulation, and attention. Neuron. 2005;46:681–692.
